# CircGLIS3 promotes gastric cancer progression by regulating the miR-1343-3p/PGK1 pathway and inhibiting vimentin phosphorylation

**DOI:** 10.1186/s12967-023-04625-2

**Published:** 2024-03-08

**Authors:** Yongxin Zhang, Xiaofeng Wang, Wenwei Liu, Tianxiang Lei, Tang Qiao, Wei Feng, Wu Song

**Affiliations:** 1 Department of Gastrointestinal Surgery, The First Affiliated Hospital, Sun Yat-sen University, Guangzhou, China; 2 Institute of Precision Medicine, The First Affiliated Hospital, Sun Yat-sen University, Guangzhou, Guangdong China; 3 Laboratory of General Surgery, The First Affiliated Hospital, Sun Yat-sen University, Guangzhou, China

**Keywords:** CircGLIS3, gastric cancer, PGK1, vimentin, macrophage

## Abstract

**Background:**

Circular RNAs (circRNAs) have been proved to play crucial roles in the development of various cancers. However, the molecular mechanism of circGLIS3 involved in gastric cancer (GC) tumorigenesis has not been elucidated.

**Methods:**

The higher expression level of circGLIS3 was identified in GC through RNA sequencing and subsequent tissue verification using Quantitative real-time PCR (qRT-PCR). A series of functional experiments in vitro and in vivo were performed to evaluated the effects of circGLIS3 on tumor growth and metastasis in GC. The interaction and regulation of circGLIS3/miR-1343-3p/PGK1 axis was confirmed by RNA pulldown, western blot, and rescue experiments. RIP and western blot were performed to demonstrate the role of circGLIS3 in regulating phosphorylation of VIMENTIN. We then used qRT-PCR and co culture system to trace circGLIS3 transmission via exosomal communication and identify the effect of exosomal circGLIS3 on gastric cancer and macrophages. Finally, RIP experiments were used to determine that EIF4A3 regulates circGLIS3 expression.

**Results:**

CircGLIS3(hsa_circ_0002874) was significantly upregulated in GC tissues and high circGLIS3 expression was associated with advanced TNM stage and lymph node metastasis in GC patients. We discovered that overexpression of circGLIS3 promoted GC cell proliferation, migration, invasion in vitro and in vivo, while suppression of circGLIS3 exhibited the opposite effect. Mechanistically, circGLIS3 could sponge miR-1343-3p and up-regulate the expression of PGK1 to promote GC tumorigenesis. We also found that circGLIS3 reduced the phosphorylation of VIMENTIN at ser 83 site by binding with VIMENTIN. Moreover, it was proven that exosomal circGLIS3 could promote gastric cancer metastasis and the M2 type polarization of macrophages. In the final step, the mechanism of EIF4A3 regulating the generation of circGLIS3 was determined.

**Conclusion:**

Our findings demonstrate that circGLIS3 promotes GC progression through sponging miR-1343-3p and regulating VIMENTIN phosphorylation. CircGLIS3 is a potential therapeutic target for GC patients.

**Supplementary Information:**

The online version contains supplementary material available at 10.1186/s12967-023-04625-2.

## Introduction

As one of the leading causes of cancer-related death worldwide, the molecular mechanisms underlying gastric cancer (GC) remain largely unknown [1]. Although the combination of surgery and chemotherapy has made great progress, the prognosis of gastric cancer still has not been significantly improved [2]. In the complex process of gastric cancer development, a variety of molecules and signaling pathways are altered [3]. Therapeutic strategies for these molecules have become promising for GC patients; thus, it is necessary to elucidate the molecular mechanism of GC to develop new biomarkers and therapeutic targets.

Circular RNAs (circRNAs) have emerged as a novel type of endogenous noncoding RNA [4] and are characterized by a covalently closed continuous loop with a back splice site [5]. Compared with their linear counterparts, circRNAs are highly stable due to their circular structure, which makes them potential diagnostic and prognostic biomarkers for various diseases, especially cancer [6]. Emerging evidence shows that multiple circRNAs are involved in the initiation and development of multiple cancers [7–9], such as proliferation, invasion and metastasis [10]. Despite several circRNAs have been reported in gastric cancer, the roles and functions of circGLIS3 in GC have not been elucidated.

The latest research shows that circRNAs are abundant in exosomes and that their expression is stable. In serum exosomes, circRNAs can promote the occurrence and progression of many tumors through RNA‒RNA competitive interactions [11, 12]. In addition, tumor-derived exosomal circRNAs have been proven to be able to induce M2 polarization of tumor-associated macrophages (TAMs) in a variety of tumors, thus mediating tumor invasion and metastasis [13–15], but are rarely reported in gastric cancer. TAMs account for 50% of the tumor mass and play an important role in tumor progression [16], including M1 and M2 types [17]. Most studies have shown that M2-polarized macrophages have anti-inflammatory effects and are related to the poor prognosis of cancer. They affect the invasion and metastasis of gastric cancer by secreting a variety of cytokines and chemokines [18]. Therefore, it is of great significance to explore the key role of exosomal circRNAs in regulating TAM polarization in the gastric cancer microenvironment.

In this study, we identified a novel GC-related circRNA termed circGLIS3 from exon 2 of the GLIS3 gene with a circBase ID of hsa_circ_0002874, which was upregulated in GC tissues, and promoted tumorigenesis and carcinogenic progression by regulating the miR-1343-3p/PGK1 pathway and inhibiting vimentin phosphorylation at Ser83. Moreover, circGLIS3 is packaged into exosomes and transmitted to neighboring gastric cancer cells and macrophages, enabling them to acquire invasive capacity and M2 macrophage properties respectively, thereby promoting the progression of gastric cancer. Our study provides new insight into the metastasis-related process and therapeutic target of GC.

## Method

### Clinical specimens and ethical approval

We collected 50 paired GC tissues and paired normal tissues from patients with gastric cancer. Who underwent gastrectomy at the Department of Gastrointestinal Surgery of First Affiliated Hospital of Sun Yat-sen University between November 2019 and December 2021. None of these Patients received preoperative chemotherapy or radiotherapy. Three paired tissues were used for RNA sequencing. All specimens were frozen in liquid nitrogen after surgical resection and stably stored in liquid nitrogen until RNA extraction. Clinicopathological features, including age, sex, differentiation grade, TNM stage (American Joint Committee on Cancer classification, AJCC), lymphatic invasion are shown in Table 1. The present study was approved by the Ethics Committee of the First Affiliated Hospital of Sun Yat-sen University. Written informed consent was obtained from patients involved in the study.

**Table 1:** Correlation between circGLIS3 expression level and clinical characteristics of gastric cancer

Parameters	Group	*circGLIS3* expression			
		Cases	Low	High	P value
Gender	Male	38	19	19	0.6145
	Female	12	7	5	
Age	< 55	15	6	9	0.4585
	≥ 55	35	18	17	
Tumor size	≤ 4 cm	18	11	7	0.3335
	> 4 cm	32	15	17	
Histology grade	Well-moderately	27	13	14	0.5547
	Poorly	23	13	10	
Tumor site	Cardiac	14	9	5	0.2782
	Non-cardiac	36	17	19	
Depth of invasion	T1 + T2	18	12	6	*0.0475
	T3 + T4	32	12	20	
Lymphatic invasion	N0	17	12	5	*0.0218
	N1-N3	33	12	21	
TNM stage	I + II	23	17	6	*0.0007
	III + IV	27	7	20	

*p<0.05

### Cell culture

The normal human gastric epithelial cell line GES-1 and GC cell lines (AGS, MKN1, SGC-7901, MKN-45, HGC-27, BGC-823) were obtained from the Laboratory of General Surgery of the First Affiliated Hospital of Sun Yat-sen University, Shanghai Zhong Qiao Xin Zhou Biotechnology Co., Ltd., Procell Life Science & Technology Co., Ltd. HGC27, BGC823 and GES1 cells were cultured in RPMI 1640 medium (Gibco, Carlsbad, CA, USA). AGS, MKN1, SGC-7901, and MKN-45 cells were cultured in Dulbecco’s modified Eagle’s medium (DMEM) (Gibco). All media were supplemented with 10% fetal bovine serum (FBS), 100 U/ml penicillin, and 100 μg/ml streptomycin (HyClone).

### RNA sequencing (RNA-seq) analysis

RNA degradation and contamination were monitored on 1% agarose gels. RNA integrity was assessed using the RNA Nano 6000 Assay Kit of the Bioanalyzer 2100 system (Agilent Technologies, CA, USA). Ribosomal RNA was removed by an Epicenter Ribozero^™^ rRNA Removal Kit (Epicenter, USA). The sequencing libraries were generated by the NEBNext^®^ Ultra^™^ Directional RNA Library Prep Kit for Illumina^®^ (NEB, USA) following the manufacturer’s recommendations. Finally, the products were purified (AMPure XP system), and library quality was assessed on the Agilent Bioanalyzer 2100 system. The clustering of the index-coded samples was performed on a cBot Cluster Generation System using TruSeq PE Cluster Kit v3-cBot-HS (Illumina) according to the manufacturer’s instructions. After cluster generation, the libraries were sequenced on an Illumina HiSeq 4000 platform, and 150 bp paired-end reads were generated.

### Plasmids and siRNA transfection and lentiviral transduction

The plasmids pLC5-ciR-circGLIS3 and pLC5-ciR-circGLIS3-MS2, miRNA mimics and inhibitors were designed and synthesized by Geneseed Biotechnology (Guangzhou, China). siRNAs targeting circGLIS3 were designed and synthesized by RiboBio (Guangzhou, China). siRNA sequences included si-hsa_circ_0002874_001 ATCCTGGGAAAGGCTTATA and si-hsa_circ_0002874_002 GAAAGGCTTATAACCCACA. The plasmids and miRNA mimics or inhibitors were transfected into cells with Lipofectamine 3000 (Invitrogen). Lentiviral transduction was produced by the Lenti-Pac™ HIV Expression Packaging Kit (GeneCopoeia). The lentivirus vector (psi-LVRU6GP/GFP/Puro) containing shRNAs targeting circGLIS3 was generated by GeneCopoeia (Guangzhou, China). Stable cell lines were obtained by selection with puromycin. psi-LVRU6GP (GeneCopoeia Biotechnology, Guangzhou, China) was then transfected into these cell lines for bioluminescence imaging.

### circRNA pull-down assays

An MS2 bacteriophage coat protein (MS2-CP) circRNA pull-down assay was performed using the MS2 tagging technique, which is based on the natural binding between a stem‒loop structure of MS2 and MS2-CP. In brief, we constructed a plasmid with circGLIS3 and MS2, which was fused with green fluorescent protein (GFP) (circGLIS3-MS2GFP). We also constructed a plasmid with MS2-CP-Flag, which was fused with an mCherry tag (MS2-CP-FlagmCherry). AGS cells were transfected with these two plasmids, and circGLIS3 was precipitated by pull down using anti-Flag antibodies. As controls, lysates derived from cells without the MS2 tagging system were used. The cell lysates were incubated with Protein A/G beads overnight at 4 °C. After washing, circGLIS3-MS2-bound proteins were eluted with urea buffer supplemented with dithiothreitol, trypsin and LysC. Next, the RNA and bound proteins were eluted with the HiPure Total RNA Mini Kit (MAGEN, Guangzhou, China). The RNA of the pulldown group and the input group were then subjected to miRNA sequencing. The bound proteins were analyzed by label-free mass spectrometry (MS).

### circRNA immunoprecipitation (circRIP) assays

The circRIP assay was performed with an RNA-Binding Protein Immunoprecipitation Kit (17–700, Merck, Millipore). We followed the manufacturer’s instructions to determine the binding between the vimentin protein (10366-1-AP, Proteintech), EIF4A3 protein (17504-1-AP, Proteintech) and circGLIS3. The procedure complied with the guidance of the manufacturer. Finally, we performed qRT‒PCR on the vimentin-associated RNA mixture absorbed by the magnetic beads.

### Western blot

For western blot analysis, total proteins were extracted using the Whole Cell Protein Extraction Kit (Key GEN, China). A BCA Protein Quantitation Assay (Thermo, USA) was used to measure the protein concentration. We separated protein samples by using 10% sodium dodecyl sulfate‒polyacrylamide gel electrophoresis (SDS‒PAGE) gels. The separated protein samples were then transferred onto a polyvinylidene fluoride (PVDF) membrane (Millipore, USA). After blocking with 5% nonfat dry milk in Tris-buffered saline (TBS)/0.1% Tween 20 for 1 h at room temperature, the membranes were incubated with AKT (1:1000, 10176-2-AP, Proteintech), PI3K (1:1000, 60225-1-Ig, Proteintech), p-PI3K (1:1000, 28731-1-AP, Proteintech), p-PI3K (1:1000, ab182651, Abcam), PGK1 (1:1000, 17811-1-AP, Proteintech), Ser82 VIMENTIN (1:1000, ab52943-10, Abcam), Ser72 VIMENTIN (1:1000, ab52944, Abcam), Ser38 VIMENTIN (1:1000, ab52942, Abcam), Ser83 VIMENTIN (1:1000, 3878 T, CST, USA) and anti-GAPDH (1:1000, 5,174, CST, USA). The next day, membranes were washed 3 times with TBST buffer for 15 min and incubated with horseradish peroxidase-conjugated secondary antibody. Finally, the western blot signals were visualized using Immobilon Western Chemiluminescent HRP Substrate (Millipore, USA).

### RNA preparation and reverse transcription-quantitative real-time PCR

All RNAs were isolated by RNA isolation plus (TaKaRa, Japan) according to the manufacturer’s protocol. cDNA was generated using PrimeScript RT Reagent (TaKaRa). The relative expression levels were measured by quantitative real-time reverse transcription polymerase chain reaction by using a LightCycler480 II Real-time PCR System (Roche, USA) with the SYBR green detection system (Takara). The samples were placed in a 96-well plate and amplified using the manufacturer’s standard amplification conditions (stage 1: 30 s at 95 °C, stage 2: 40 cycles of 5 s at 95 °C and 34 s at 60 °C, stage 3: melt curve). Relative expression was determined by the 2^−ΔΔCT^ method. Meanwhile, we used GAPDH as an endogenous control for mRNA. The primer sequences used were as follows: hsa_circ_0002874_ divergent (forward: 5′- AGGAGACAAATGCTCACCAATG -3′, reverse: 5′- CATGGTGTGGGTTATAAGCCTTT-3′); hsa_circ_0002874_ convergent (forward: 5′- TCACATTCCTGCCATCCGAG-3′, reverse: 5′-TGTTAGCAAGGCTTGCCATAGT-3′); GAPDH (forward: 5′CAAGGTCATCCATGACAACTTTG-3′, reverse: 5′-GGCCATCCACAGTCTTCTGG-3′); PGK1 (forward: 5′GACCTAATGTCCAAAGCTGAGAA-3′); reverse: 5′-CAGCAGGTATGCCAGAAGCC-3′).

### RNA fluorescence in situ hybridization (FISH) and immunofluorescence analysis (IF)

Cy3-labeled probes specific for circGLIS3 were designed and synthesized by GenePharma, and the signals were detected by a FISH Kit (GenePharma) according to the manufacturer’s instructions. FISH + IF was performed with a FISH in situ hybridization immunofluorescence counterstaining kit (C009, Gefan, Shanghai). Confocal images were captured using Zeiss AIM software and a Zeiss LSM 880 with Airyscan confocal microscope system (Carl Zeiss Jena, Oberkochen, Germany).

### CCK-8

The proliferation rate of AGS/HGC27 cells was detected by the Cell Counting Kit-8 assay (Dojindo Laboratories, Kumamoto, Japan). Ten thousand cells were seeded in a 96-well plate, and 10 μL of CCK-8 solution was added to each well at the same time every day. After a 2 h incubation, the absorbance at 450 nm of the experimental wells was measured with Sunrise (Tecan, GmbH Untersbergstrasse, Austria).

### Transwell assay

The methods used for the migration assay and invasion assay were similar. We placed transwell assay inserts (Millipore, Billerica, MA, USA) in a 24-well plate. However, the difference between the methods lies in the type of membrane: The membrane in the upper transwell chamber for the invasion assay was a Matrigel-coated membrane (BD Biosciences), while that for the migration assay was a normal membrane. In the experiment, we first placed 500 μl of serum-free RPMI 1640 with 10% FBS in the bottom chamber. Next, we seeded 10,000 cells in 200 μl of RPMI 1640 in the upper chamber. After 24–48 h, we used methanol to fix the cells within the membrane and stained them with crystal violet. Finally, the cells were observed by microscopy.

### 5-Ethynyl-2′-deoxyuridine (EdU) incorporation assay

EdU assays were performed with a Cell-Light EdU DNA Cell Proliferation Kit (RiboBio). Cells were seeded at 30% confluence in 6-well plates after 48 h of transfection and were continuously cultured for 24 h. After incubation with 50 μM EdU for 2 h, the cells were fixed in 4% paraformaldehyde and stained with Apollo Dye Solution. Hoechst 33,342 was used to stain the nucleic acids within the cells. Images were obtained with a Nikon Ti microscope (Nikon, Tokyo, Japan), and the number of EdU-positive cells was counted.

### Exosome isolation and identification

Exosomes from cells were collected from 20 ml of culture media (1 × 107 cells). The media were collected on ice, centrifuged at 800 × g for 10 min to sediment the cells, and then centrifuged at 12,000 × g for 30 min to remove the cellular debris. Exosomes were separated from the supernatant by centrifugation at 100,000 × g for 2 h in a Type 70Ti rotor (Beckman Coulter). The exosome pellet was washed once in a large volume of PBS and resuspended in 100 μl of PBS. Exosomes were then identified by transmission electron microscopy (TEM) (Philips TECNAI 20, Netherlands) for particle size and form. Exosome protein markers were identified by western blot assay. The total amount of exosomes was detected by nanoparticle tracking analysis (NTA). The RNA in the exocrine body was quantified with an external reference according to the external standard kit (λ PolyA) for qPCR [19, 20].

### Animal models

Stably transfected BGC-823 cells (3 × 10^6^ cells) were subcutaneously injected into 4- to 6-week-old female BALB/c nude mice (BesTest Bio-Tech Co. Ltd, Zhuhai, China). The tumor volumes were measured once every 3 days and calculated using the following formula: volume = (length x width2)/2. After 28 days, the mice were sacrificed, and then tumors were dissected and weighed. The animal procedures were approved by the Research Ethics Committee of The First Affiliated Hospital of Sun Yat-sen University.

### Statistics

We performed our experiments in triplicate, and the results are presented as the mean value ± standard deviation. We statistically analyzed the data with Student’s t test using SPSS statistical software, and p < 0.05 was considered statistically significant. * indicates p < 0.05, ** indicates p < 0.01 and *** indicates p < 0.001.

## Result

### Identification of high expression and circular characteristics of circGLIS3 in gastric cancer

**Fig. 1:**
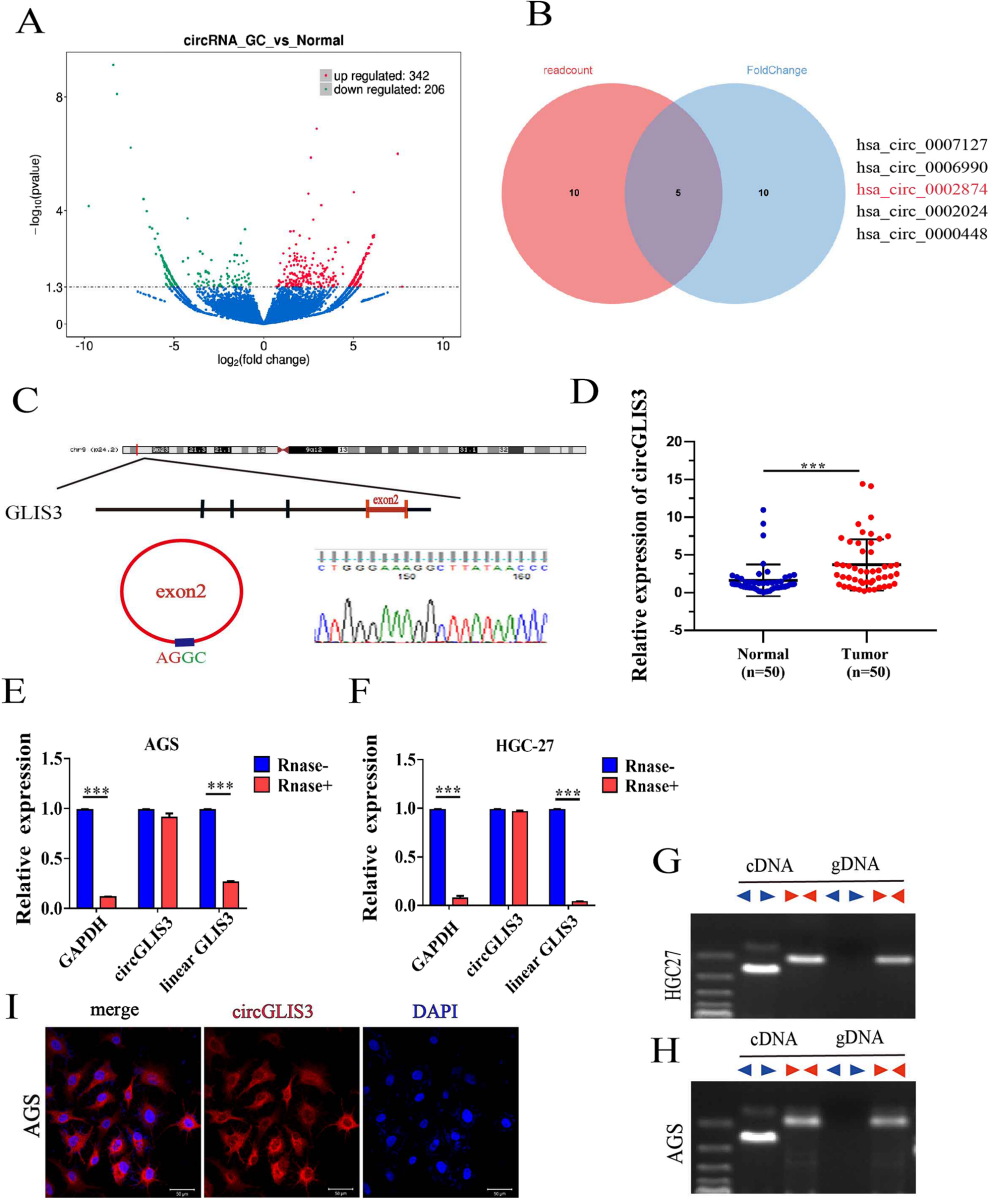
The expression of circGLIS3 in gastric cancer is elevated and related to disease progression. **A**. Volcanic maps identify differentially expressed circRNAs of gastric cancer and adjacent tissues. **B**. Significantly upregulated circRNAs identified using fold change and read count via Venn diagram. **C**. Circular characteristic structure and parental gene location. **D**. Identification of the high expression of circGLIS3 in gastric cancer tissues by qRT‒PCR. **E**, **F**. The expression of GAPDH, linear parent gene and circGLIS3 after RNase digestion using RT‒qPCR. **G**, **H**. DNA electrophoresis was used to detect the amplification of cDNA and gDNA with convertor and divergent primers. **I**. The cellular localization of circGLIS3 using FISH. * P < 0.05, **P < 0.01, ***P < 0.001

We performed RNA-seq analysis using 3 gastric cancer tissues and their paired adjacent normal tissues. A total of 342 upregulated and 206 downregulated circRNAs were identified (Fig. 1A). We performed GO and KEGG analysis of the host genes based on differentially expressed circRNAs, and the results revealed that these genes were enriched in the PI3K-Akt signaling pathway, pathways in cancer, and the Wnt signaling pathway (Additional file 1: Fig S1A, B). After tissue verification of several circRNAs based on read count and fold change using qRT‒PCR, circGLIS3 was selected for further research (Fig. 1B, Additional file 2: Table S1). In 50 paired GC samples, the expression of circGLIS3 relative to adjacent normal samples was significantly increased, which was consistent with the RNA-seq data (Fig. 1D). The correlation between the expression of circGLIS3 in gastric cancer tissues and clinicopathological characteristics is listed in Table 1. The high expression of circGLIS3 was associated with advanced GC (stage III + IV), lymph node metastasis, and depth of invasion.

CircGLIS3 is derived from the GLIS3 gene, located on chromosome 9, and consists of head-to-tail splicing of exon 2, chr9:4286037–4286523. Subsequently, we confirmed the head-to-tail splicing of circGLIS3 in the RT‒PCR product of circGLIS3 by Sanger sequencing, which was consistent with the CircBase database (Fig. 1C). We also performed qRT‒PCR analysis using specially designed divergent and convergent primers and found that circGLIS3, but not linear GLIS3 or GAPDH, can resist RNase R digestion (Fig. 1E, F). In addition, we used PCR and agarose gel electrophoresis to detect the expression level of back-spliced or canonical forms of GLIS3 in the cDNA and gDNA of GC cells. circGLIS3 could not be amplified in gDNA by divergent primers, which proved that circGLIS3 has the characteristic back-spliced structure of circular RNA (Fig. 1G, H). FISH localization experiments using AGS and HGC27 cells showed that circGLIS3 was mainly localized in the cytoplasm, and a small amount was located in the nucleus (Fig. 1I). To summarize, circGLIS3 was confirmed to be a circular RNA and involved in the clinical progression of gastric cancer.

### CircGLIS3 plays a tumor-promoting role in GC growth in vivo and in vitro

**Fig. 2:**
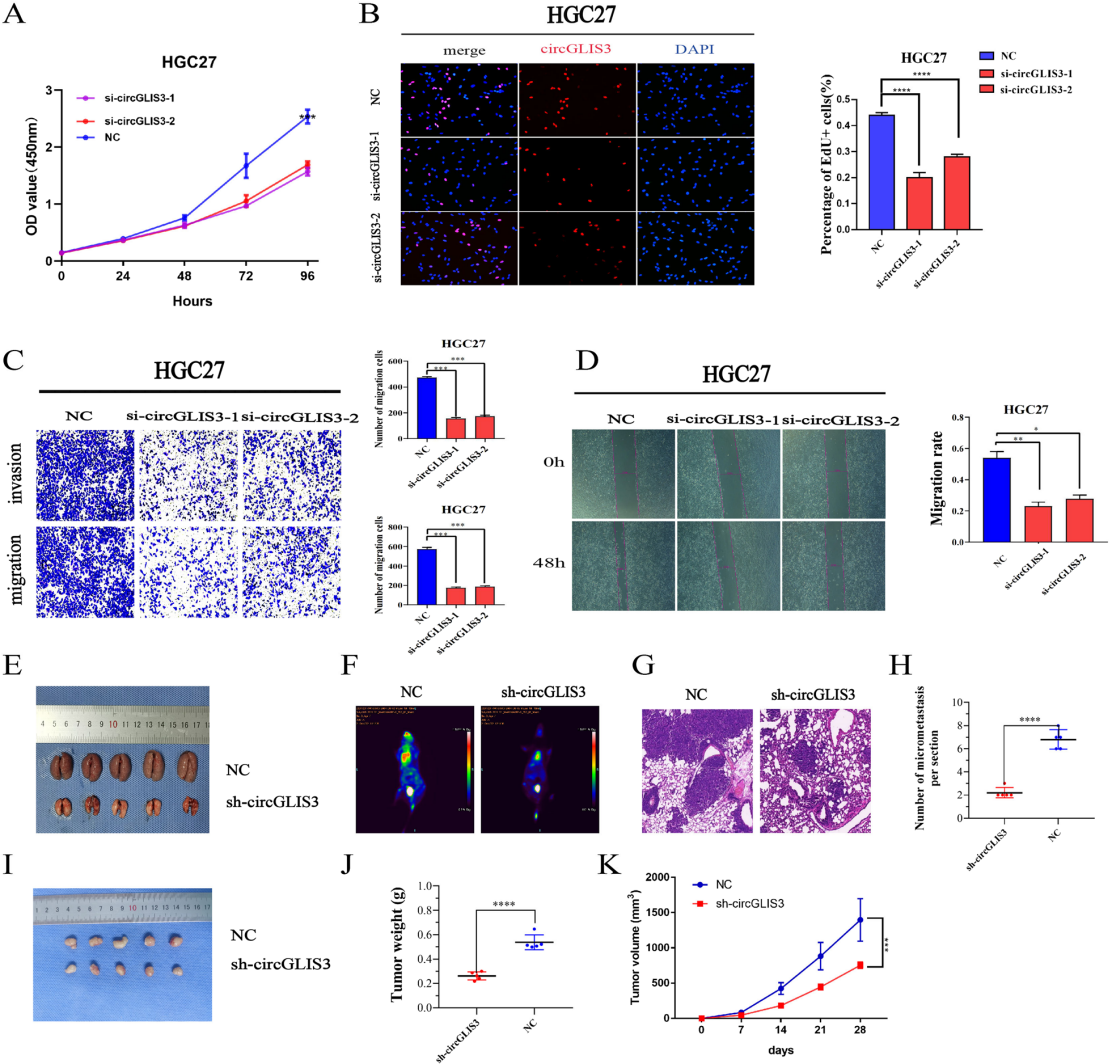
The role of circGLIS3 in promoting the growth, proliferation, invasion and metastasis of gastric cancer in HGC27 cells. **A**, **B**. CircGLIS3 silencing significantly inhibited the cell proliferation rate, as indicated by the EdU assay and the CCK8 assay. **C**, **D**. Knockdown of circGLIS3 successfully reduced the migration and invasion ability of GC cells, as determined by the Transwell experiment and wound healing assay. E.F.G.H. Nude female mice were injected with 2 × 10^6^ stably knocked down circGLIS3 and the corresponding control GC cells through the tail vein, and lung metastasis in vivo was evaluated using live imaging combined with HE staining. **I**–**K**. Nude female mice were subcutaneously injected with 5 × 10^6^ stably knocked down circGLIS3 and the corresponding control GC cells, and the tumors were extracted after 21 days. *P < 0.05, **P < 0.01, ***P < 0.001, ****P < 0.0001

We selected some gastric cancer cell lines and GES-1 cell lines to detect the expression level of circGLIS3 and then selected HGC27 and AGS for functional experiments (Additional file 1: Fig S1C). HGC-27 cells showed the highest expression of circGLIS3, and AGS and BGC-823 cells showed the second and third highest expression of circGLIS3, respectively. Therefore, we selected HGC-27, AGS and BGC-823 GC cells for the follow-up study of circGLIS3. To further explore the functional effects of circGLIS3 on GC cells, we designed two siRNA and overexpression plasmids and verified their efficiency (Additional file 1: Fig S1D–G). CCK-8 and EdU assays were used to verify the proliferation ability of GC cells when circGLIS3 was knocked down in gastric cancer cells (Fig. 2A, B). In addition, cell migration was assessed by Transwell and wound healing assays, and circGLIS3-specific siRNA inhibited cell migration and invasion (Fig. 2C, D). Overexpression of circGLIS3 had the opposite effect, enhancing proliferation (Additional file 1: Fig S2A, B), cell migration and invasion (Additional file 1: Fig S2C, D). We transfected the circGLIS3 knockdown plasmid into the BGC-823 cell line to achieve a stable knockdown effect (Additional file 1: Fig S1H). The subcutaneous xenotransplantation experiment and tail vein pulmonary metastasis experiment confirmed that circGLIS3 can promote the invasion, metastasis (Fig. 2E–H), growth and proliferation (Fig. 2I–K) of gastric cancer cells in vivo. Next, we constructed a BGC-823 cell line stably overexpressing circGLIS3 and verified the overexpression efficiency (Additional file 1: Fig S1I). We also carried out a series of in vivo experiments to confirm the role of circGLIS3 in promoting tumor metastasis (Additional file 1: Fig S2E–H) and progression (Additional file 1: Fig S2I–K). In conclusion, it is suggested that CircGLIS3 can promote tumor progression in vivo and in vitro.

### CircGLIS3 serves as a miRNA sponge of miR-1343-3p

**Fig. 3:**
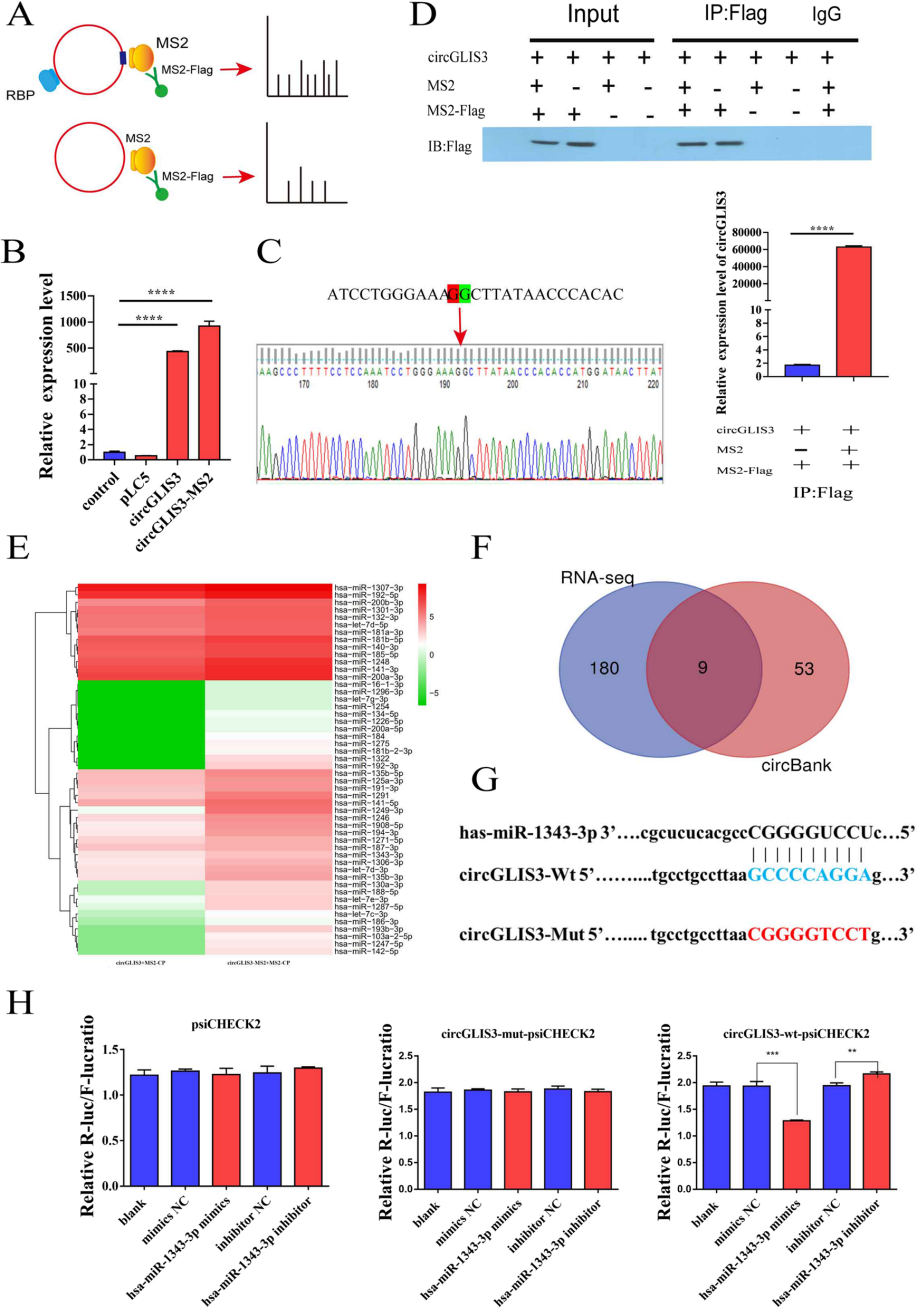
Identification of downstream miRNA molecules by circGLIS3 pull-down experiment. **A**. Schematic diagram of the circGLIS3 pull-down method using the MS2-tagging system. **B**. Confirmation of the overexpression of circGLIS3 using qRT‒PCR. **C**. PCR and Sanger sequencing were used to verify the circular characteristics. **D**. Western blot analysis of MS2-CP-Flag pulled down by anti-Flag (up). The enrichment of circGLIS3 in the complex with MS2-CP-Flag was measured by qRT‒PCR (bottom). **E**. Heatmap of the miRNAs pulled down by circGLIS3. **F**. Venn diagram shows the intersecting miRNAs pulled down by circGLIS3 and predicted by circBank based on the binding sites to circGLIS3. **G**. CircGLIS3 mutation and wild-type vector construction. **H**. A dual-luciferase reporter assay to determine the direct binding between circGLIS3 and miR-1343-3p based on their complementary sequences

To determine the mechanism and pathway of circGLIS3 in gastric cancer cells, we carried out circRNA pull-down using the MS2-tagging system (Fig. 3A). First, the overexpression of circGLIS3 in the circGLIS3-MS2 tagging system was confirmed using qRT‒PCR analysis (Fig. 3B). Sanger sequencing confirmed the junction site, which indicated that this design did not impair the circularization of circGLIS3 (Fig. 3C). We constructed two plasmids expressing circGLIS3-MS2^GFP^ and MS2-Flag^mCherry^ (Additional file 1: Fig S3A). CircGLIS3-MS2^GFP^ and MS2-Flag^mCherry^ were cotransiently transferred into AGS cells, and MS2-Flag specifically combined with MS2-labeled circGLIS3 to form the MS2-Flag-MS2-circGLIS3 complex. MS2-Flag was detected using western blot analysis with anti-Flag antibodies (Fig. 3D, top panel), and circGLIS3 enrichment following capture was confirmed through qRT‒PCR analysis (Fig. 3D, bottom panel), which demonstrated the specificity of pull-down isolation. The pull-down products were used for label-free MS analysis and miRNA sequencing. We detected that 189 miRNAs were enriched in the circGLIS3-MS2 + MS2-Flag group compared with the circGLIS3 + MS2-Flag group (Fig. 3E) and identified potential downstream pathways of these miRNAs through ceRNA network and KEGG analysis (Additional file 1: Fig S3B, C). We then took the intersection with the miRNA data predicted by the circBank database to narrow the range of possible target molecules (Fig. 3F; Additional file 3: Table S2, Additional file 4: Table S3). Among these miRNAs, we selected miR-1343-3p for validation, considering that miR-1343-3p had the highest prediction score and miRNA-seq read count. The expression level of miR-1343-3p was decreased in cells treated with circGLIS3 overexpression plasmids, which proved that miR-1343-3p is the downstream target of circGLIS3 (Additional file 1: Fig S3D). Next, we constructed circGLIS3 mutant and wild-type vectors and performed double luciferase reporter analysis to determine the direct binding between circGLIS3 and miR-1343-3p according to their complementary sequences (Fig. 3G). In the cotransfection of miR-1343-3p and circGLIS3 wild-type plasmids, the luciferase activity of the miR-1343-3p mimic group was significantly lower than that of the mimic NC group, and the luciferase activity of the miR-1343-3p inhibitor group was significantly higher than that of the mimic NC group. However, no significantly increased or decreased luciferase activity was observed in the cotransfection of miR-1343-3p and circGLIS3 mutant plasmids (Fig. 3H). Therefore, the direct interaction of circGLIS3 and miR-1343-3p was confirmed. Based on these results, we speculated that circGLIS3 may function as a miR-1343-3p sponge.

### The circGLIS3-miR-1343-3p-PGK1 axis promotes GC development

**Fig. 4:**
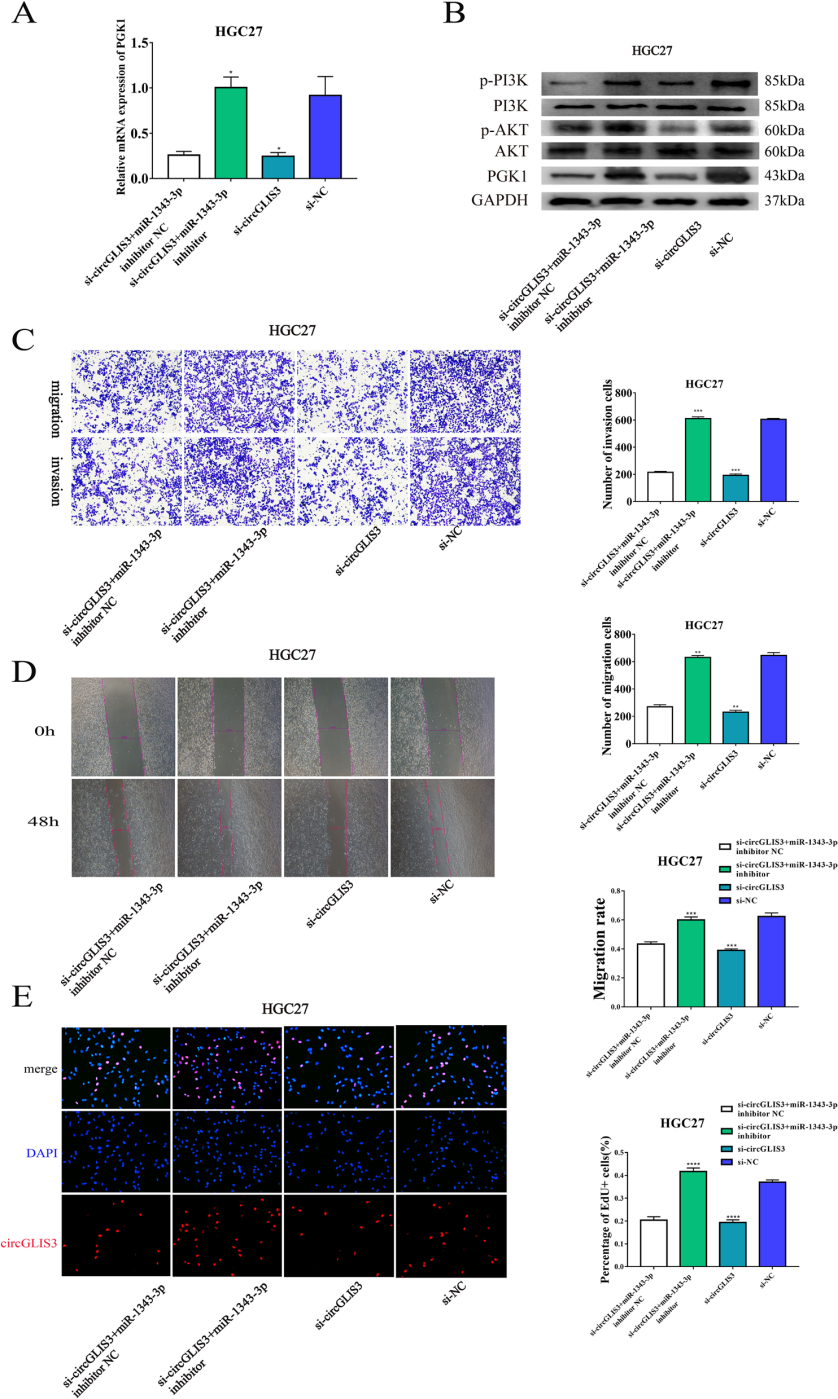
Verification of the circGLIS3/miR-1343-3p/PGK1 axis by the HGC27 cell line in a rescue experiment. **A**, **B**. The expression levels of PGK1 mRNA and protein and the AKT signaling pathway were measured by qRT‒PCR and WB after simultaneous knockdown of circGLIS3 and miR-1343-3p in the HGC27 cell line. Cotransfection of miR-1343-3p inhibitor and circGLIS3 siRNA to investigate malignant transformation of cells with Transwell (**C**), wound healing (**D**), and EdU assays (**E**) in the HGC27 cell line* P < 0.05, **P < 0.01, ***P < 0.001, ****P < 0.0001

We conducted KEGG analysis to determine the potential target gene of miR-1343-3p using multiple databases (Additional file 5: Table S4 and Additional file 6: Table S5). The results showed that the PI3K/AKT signaling pathway was significantly enriched (Additional file 1: Fig S3E). Using the ENCORI database, we determined that PGK1 is the downstream target of miR-1343-3p, considering that PGK1 has a high target prediction score (Additional file 1: Fig S3F; Additional file 7: Table S6). MiR-1343-3p plays a tumor suppressive role in a variety of tumors, including gastric cancer [21, 22], and the interaction between miR-1343-3p and PGK1 has also been reported [23], which is consistent with our research. To determine whether PGK1 is the downstream target of circGLIS3, we knocked down circGLIS3 in a gastric cancer cell line and detected the expression level of PGK1 through western blotting. We selected si-circGLIS3-1 for further experiments based on its higher knockdown efficiency. Compared with that in the NC group, the expression level of PGK1 was decreased significantly in the circGLIS3 knockdown group (Additional file 1: Fig S3G). Thus, we proved the positive regulation of PGK1 by circGLIS3. To verify whether circGLIS3 plays the role of tumor promoter via PGK1 by sponging miR-1343-3p, we carried out a rescue experiment. MiR-1343-3p inhibitor was used to check whether the tumor suppression effect of circGLIS3 knockdown could be reversed by knocking down miR-1343-3p. Subsequently, we found that knockdown of circGLIS3 and miR-1343-3p significantly reversed the downregulation of PGK1 expression at the mRNA and protein expression levels in HGC-27 cells related to circGLIS3 inhibition (Fig. 4A). It has been reported that PGK1 can promote tumor progression through the PI3K/AKT signaling pathway. Therefore, we detected the activation of the PI3K/AKT signaling pathway at the same time and found that the miR-1343-3p inhibitor could rescue the effect of circGLIS3 inhibition on inactivating the PI3K/AKT signaling pathway (Fig. 4B). We also tried to determine whether the biological function of circGLIS3 in GC cells can be reversed by miR-1343-3p inhibitors. We observed that the inhibitory effect of circGLIS3 knockdown in the Transwell, wound healing and EdU experiments was rescued by the miR-1343-3p inhibitor (Fig. 4C–E). In addition, we carried out simultaneous overexpression of circGLIS3 and miR-1343-3p mimics in AGS cell lines and detected the expression levels of PGK1 and PI3K/AKT signaling pathway proteins and a series of functional experiments (Additional file 1: Fig S4A–E). The results showed that the effect of circGLIS3 overexpression on tumor progression was abolished by miR-1343-3p mimics, following changes in the PGK1 and PI3K/AKT signaling pathways. Overall, we proved that circGLIS3 promotes GC progression as a sponge of miR-1343-3p through the PGK1-mediated PI3K/AKT pathway.

### CircGLIS3 decreases phosphorylation of VIMENTIN at the Ser83 site by binding VIMENTIN

**Fig. 5:**
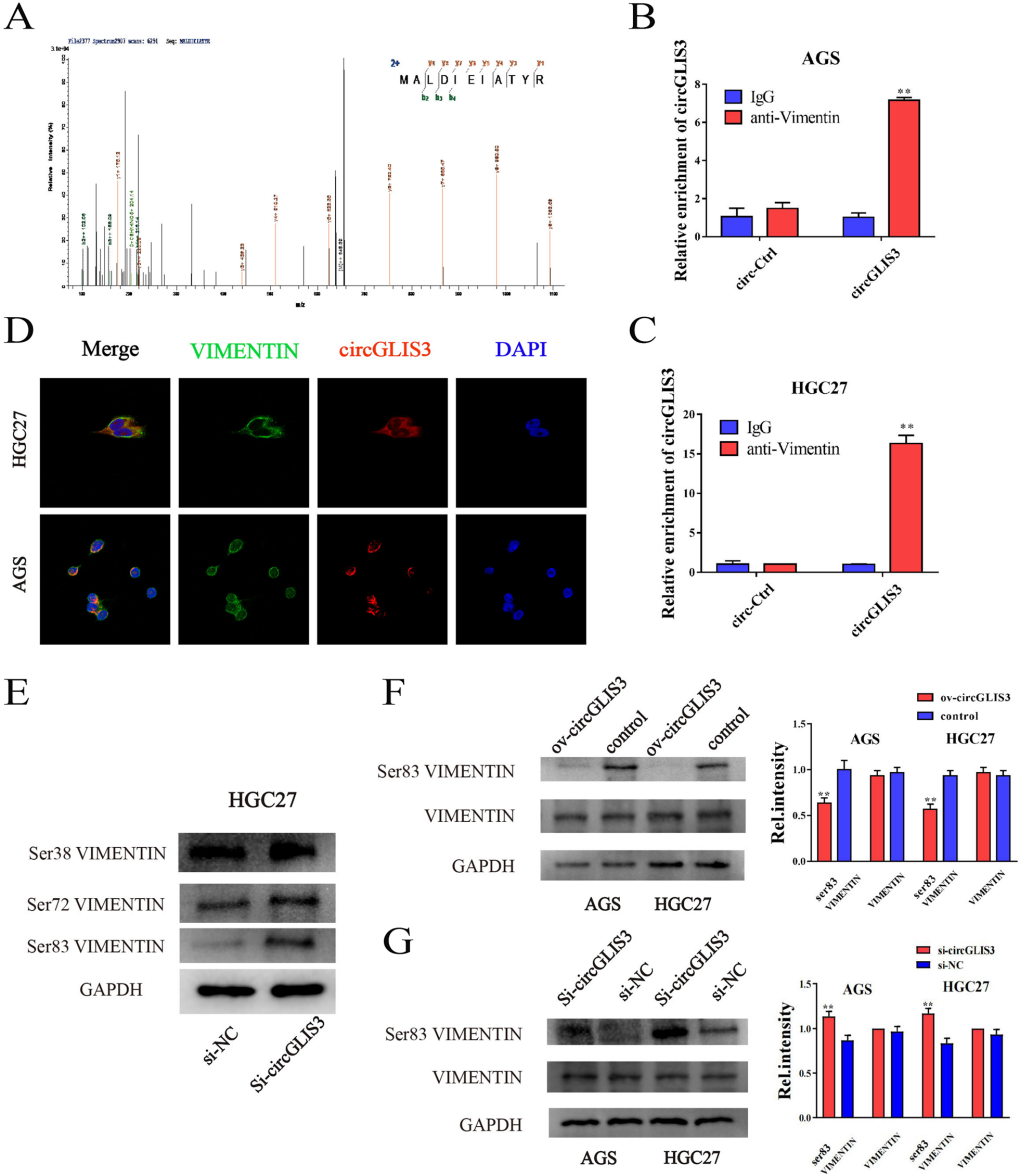
CircGLIS3 downregulates phosphorylation of VIMENTIN at Ser83. **A**. The protein complex with MS2-CP-Flag was tested using label-free MS. The peptides were matched to VIMENTIN. **B**, **C**. circRNA immunoprecipitation (circRIP) assay to measure the amount of circGLIS3 pulled down by VIMENTIN and IgG antibodies in AGS and HGC27 cells stably overexpressing circGLIS3 and circ-Ctrl. **D**. Colocalization of VIMENTIN protein and circGLIS3 in HGC27 and AGS cell lines. **E**. Changes in VIMENTIN phosphorylation levels at Ser38, Ser72 and Ser83 in the circGLIS3 knockdown group compared with the NC group were observed. **F**, **G**. After overexpression of circGLIS3, the phosphorylation level of the VIMENTIN Ser83 site was significantly reduced, while knockdown of circGLIS3 showed the opposite effect. * P < 0.05, **P < 0.01

We also detected the peptides of proteins bound to circGLIS3 and matched peptides to VIMENTIN (Fig. 5A, Additional file 8: Table S7). These data validated the interaction between circGLIS3 with vimentin. To further verify the binding of circGLIS3 to VIMENTIN, a circRIP assay was conducted in AGS and HGC27 cells using anti-VIMENTIN antibodies. The expression of circGLIS3 was enriched after anti-vimentin immunoprecipitation compared with IgG (Fig. 5B, C). We also found that VIMENTIN was colocalized with circGLIS3 in the cytoplasm, which further suggested an interaction between circGLIS3 and VIMENTIN (Fig. 5D). Studies have reported that phosphorylation of VIMENTIN at different sites affects the invasion and metastasis ability of tumor cells [24]. We selected Ser38, Ser72 and Ser83 of VIMENTIN phosphorylation sites for study and detected the changes in these sites after knockdown of circGLIS3 using Western blotting. The results showed that Ser38 and Ser72 had no significant changes, while Ser83 was significantly increased in the circGLIS3 knockdown group (Fig. 5E). Extended validation in AGS and HGC27 cell lines showed that compared with the control group, the Ser83 phosphorylation level in the circGLIS3 overexpression group was significantly reduced (Fig. 5F), while compared with the NC group, Ser83 in the circGLIS3 knockdown group was significantly increased (Fig. 5G). Thus, we proved that circGLIS3 can reduce the phosphorylation of vimentin Ser83 to promote the invasion and metastasis of gastric cancer.

### Exosomal circGLIS3 regulates PGK1 expression and vimentin Ser83 phosphorylation in vitro and promotes metastasis in vivo

**Fig. 6:**
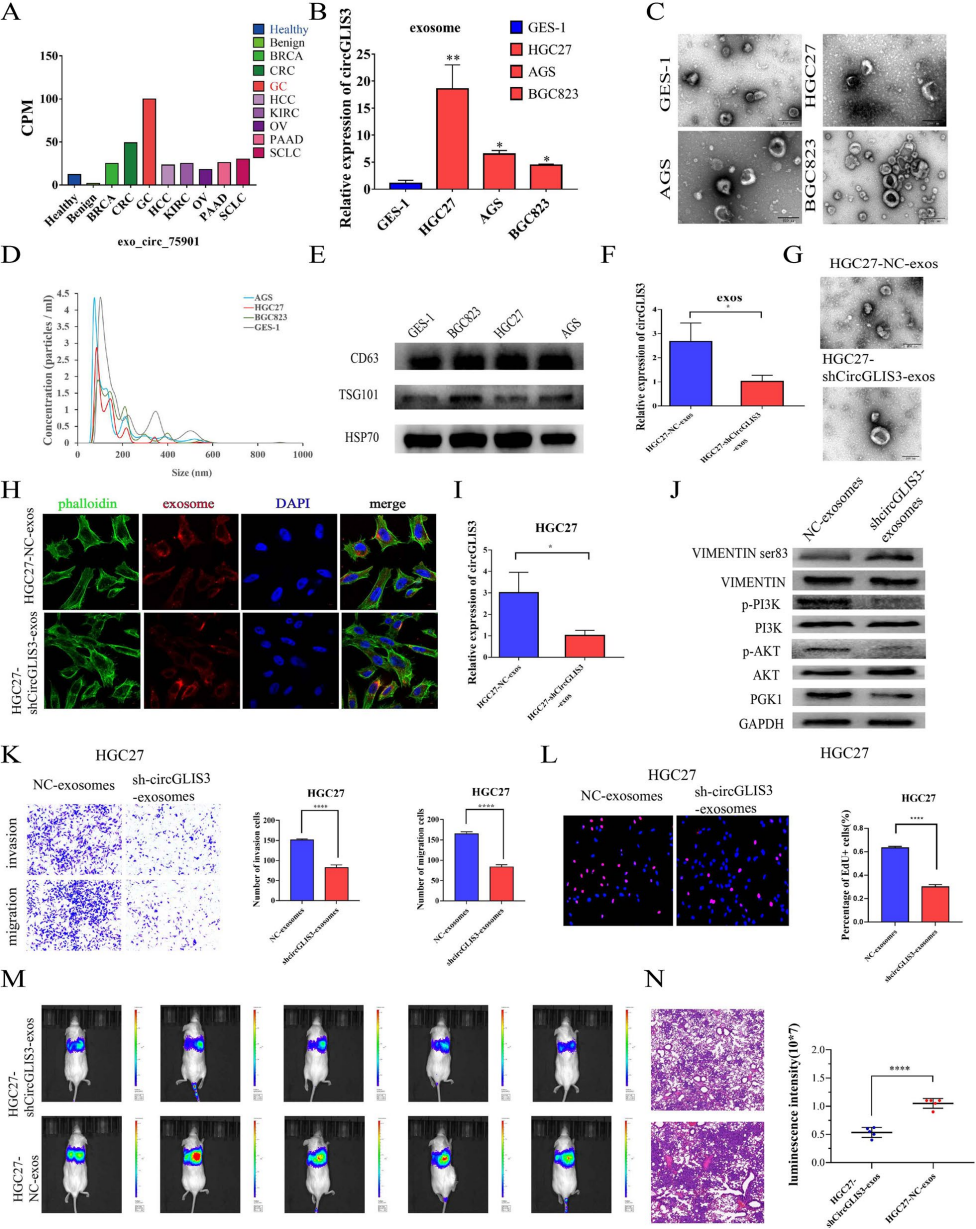
Exosomal circGLIS3 promotes the progression of gastric cancer. **A**. CircGLIS3 in serum exosomes of gastric cancer patients was significantly higher than that of healthy people according to the exoRBase database. **B**. qPCR analysis of the expression levels of circGLIS3 in the culture medium (CM) of different GC cell lines and one normal gastric cell line. **C**, **D**. Phenotype analysis of exosomes derived from HGC27, AGS, BGC823, and GES-1 cells using electron microscopy and Nano Sight nanoparticle tracking analysis. **E**. Western blot analysis was performed to detect typical exosomal biomarkers (TSG101, CD63, and HSP70) in exosomes derived from the above cell lines. **F**. qRT‒PCR was used to verify the expression of circGLIS3 in the exosomes of circGLIS3-knockdown GC cells and NC cells. **G**. We used a transmission electron microscope (TEM) to determine the existence and morphology of exosomes purified from GC cell medium (exosome-free FBS). **H**. Red exosome signals were found in the cytoplasm of GC cells when purified exosomes were added to phalloidin-labeled GC cells for 72 h. **I**. We detected lower circGLIS3 expression in GC cells by coculturing them with exosomes from circGLIS3-knockdown GC cells compared with NC cells for 72 h via qRT‒PCR. J. WB was used to verify the changes in PGK1, p-AKT, p-PI3K, and vimentin Ser83 in GC cells after coculturing them with exosomes purified from circGLIS3-knockdown GC cells relative to those from NC cells. **K**, **L**. We found that GC cells treated with circGLIS3-knockdown exosomes showed lower metastasis and proliferation potential. M.N. Nude female mice were injected with exosomes purified from circGLIS3-knockdown and corresponding control GC cells through the tail vein, and lung metastasis in vivo was evaluated using live imaging combined with HE staining. * P < 0.05, **P < 0.01, ***P < 0.001, ****P < 0.0001

Studies have shown that circRNAs can be secreted and packaged into exosomes to mediate communication between tumor cells. We searched circGLIS3 using the exoRBase database, and the results showed that circGLIS3 (exo_circ_75901) was significantly increased in GC patient serum (Fig. 6A). We extracted the supernatant exosomes of gastric cancer and GES-1 cell lines by ultracentrifugation and identified the extracted exosomes by electron microscopy, particle size analysis and WB (Fig. 6C–E). We next confirmed that the level of circGLIS3 in the supernatant exosomes of HGC-27, BGC-823 and AGS GC cell lines was higher than that of GES-1 cells (Fig. 6B). These results proved that exosomal circGLIS3 was enriched in GC cells.

To further validate the effect of exosomal circGLIS3 on gastric cancer cell communication, we first extracted exosomes from the circGLIS3 knockdown group and NC group. We detected lower circGLIS3 expression in exosomes purified from circGLIS3 knockdown GC cells relative to those from NC cells (Fig. 6F, G). Next, we added exosomes to gastric cancer cells and confirmed that exosomal circGLIS3 could be absorbed by gastric cancer cells using fluorescence staining. We detected lower circGLIS3 expression in GC cells by coculturing them with exosomes of circGLIS3-knockdown GC cells compared with NC cells via qRT‒PCR (Fig. 6H, I). We also detected changes in PGK1, PI3K, AKT, vimentin and related phosphorylation levels in gastric cancer cells after coculturing circGLIS3-knockdown exosomes and NC exosomes. The results showed that the expression of PGK1 was significantly decreased, the phosphorylation of PI3K and AKT was significantly decreased, and the phosphorylation of vimentin Ser83 was significantly increased (Fig. 6J), which was consistent with our previous verification of the intracellular role of circGLIS3. Subsequently, functional experiments confirmed that exosomal circGLIS3 could promote the proliferation, invasion and metastasis of gastric cancer cells (Fig. 6K, L). Recent reports have described the positive correlation between exosomal communication and metastasis. We attempted to examine the role of exosomal circGLIS3 in distant metastasis by tail vein injection of GC cells cocultured with circGLIS3-knockdown exosomes and NC exosomes into NSG mice. Based on the luciferase intensity and H&E staining detected in lung tissue, we found that GC cells treated with circGLIS3-knockdown exosomes had a lower metastatic potential (Fig. 6M, N).

We further conducted a series of experiments by extracting circGLIS3-overexpressing exosomes and control exosomes (Additional file 1: Fig S5A, B). Compared with that in the control group, the expression of circGLIS3 in gastric cancer cells increased after coculture with circGLIS3-overexpressing exosomes (Additional file 1: Fig S5C, D). At the same time, the expression level of PGK1 increased, the phosphorylation of PI3K and AKT increased, and the phosphorylation of vimentin Ser83 decreased (Additional file 1: Fig S5E). Functional experiments also showed the same trend of exosomal circGLIS3 promoting proliferation, invasion and metastasis (Additional file 1: Fig S5F, G). We conducted an in vivo experiment to demonstrate that GC cells treated with circGLIS3-overexpressing exosomes had more metastases than GC cells treated with NC exosomes (Additional file 1: Fig S5H, I). These results all proved that exosomal circGLIS3 could promote GC progression.

### Exosomal circGLIS3 induces macrophage M2 polarization to promote the invasion and metastasis of gastric cancer

**Fig. 7:**
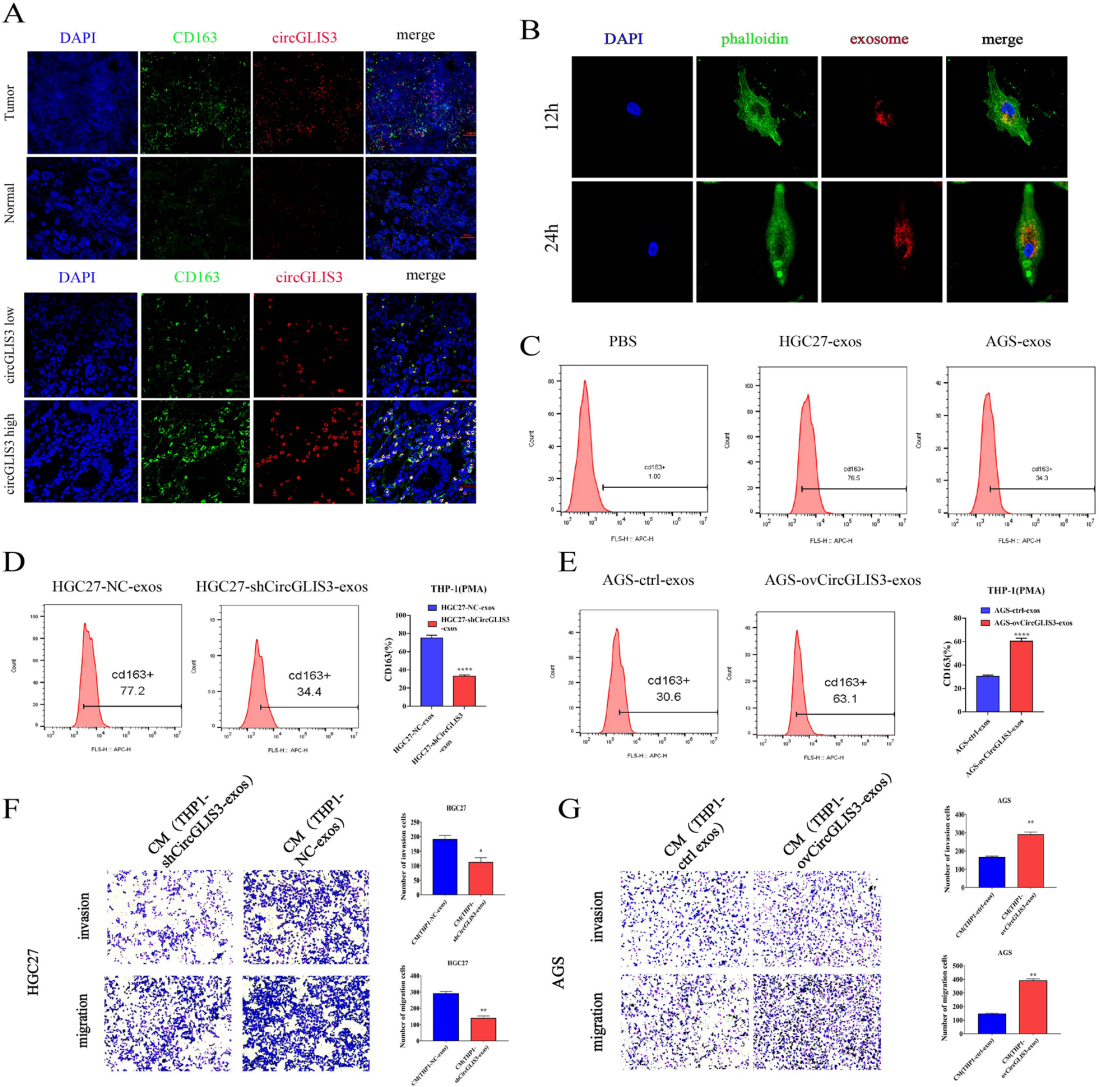
Exosomal circGLIS3 induces macrophage M2 polarization to promote the invasion and metastasis of gastric cancer. **A**. circGLIS3 and M2 macrophages were enriched and colocalized in gastric cancer compared with normal mucosa. **B**. Representative immunofluorescence image showing the internalization of DiO-labeled gastric cancer-derived exosomes (red) by PMA-treated THP-1 cells. **C**. Flow cytometry was performed to analyze the effect of GC cell-derived exosomes on the expression of the typical M2 marker CD163 in PMA-treated THP-1 cells. **D**, **E**. Flow cytometry was used to determine the effect of exosomes derived from circGLIS3-knockdown and circGLIS3-overexpressing GC cells on the expression of CD163 in PMA-treated THP-1 cells. **F**, **G**. Gastric cancer cells cocultured with CM from THP-1 cells pretreated with circGLIS3-knockdown/circGLIS3-overexpression exosomes had decreased/increased numbers of migratory and invasive cells compared to the control cells

Studies have reported that Exo-circRNA may regulate stromal cells in the microenvironment, such as macrophages, to promote gastric cancer metastasis [25–27]. Considering that circGLIS3 was highly expressed in gastric cancer exosomes and that exosomes were communication messengers between cells, we suspected that circGLIS3 may affect the state of cells in the microenvironment to promote gastric cancer metastasis. We first verified that circGLIS3 and M2 macrophages (CD163 +) were highly expressed in gastric cancer tissues compared with adjacent normal tissue. We next demonstrated that M2-type macrophages were significantly enriched in gastric cancer tissues with high circGLIS3 expression (Fig. 7A). These results suggest that gastric cancer cells may induce the transformation of macrophages into the M2 type by secreting exosomal circGLIS3. To further explore whether exosomal circGLIS3 could induce M2 polarization of macrophages, we employed the human THP-1 cell line as a normal mononuclear macrophage line. THP-1 cells were incubated with PMA to induce differentiation into M0 macrophages. We also used CM-Dil to label gastric cancer exosomes and performed exosome uptake experiments by using THP-1-induced M0 macrophages. The results showed that an increase in exosome uptake was observed at 12 h and 24 h over time, suggesting that macrophages could take up exosomes derived from gastric cancer cells (Fig. 7B). The expression of the M2 markers (CD163) was significantly increased in macrophages incubated with exosomes derived from gastric cells compared to PBS, which proved the effect of exosomes derived from gastric cancer on promoting M2 polarization of macrophages (Fig. 7C). When M0 macrophages were incubated with exosomes from cells exhibiting low circGLIS3 expression compared with the NC group, the M2 macrophage marker (CD163) was significantly decreased (Fig. 7D). Consistent with the above results, the expression levels of M2 markers were also significantly increased in macrophages treated with exosomes derived from GC cells with high circGLIS3 expression compared with the control group (Fig. 7E), demonstrating that exosomal circGLIS3 can promote the M2-type polarization of macrophages. Many studies have shown that M2-polarized macrophages promote the invasion and metastasis of gastric cancer and are associated with poor prognosis. We conducted coculture and transwell experiments to determine whether exosomal circGlIS3-polarized M2 macrophages promoted the invasion and metastasis of gastric cancer cells. In vitro transwell assays showed that HGC-27 cells cocultured with CM from THP-1 cells pretreated with circGLIS3-knockdown exosomes had decreased numbers of migratory and invasive cells compared to the control cells (Fig. 7F). In vitro transwell assays demonstrated that the numbers of migratory and invasive AGS cells in groups administered CM from THP-1 cells stimulated with PMA, which were treated with exosomes derived from circGLIS3-overexpressing GC cells, were significantly increased compared with those in the control groups (Fig. 7G). These results indicated that the M2 macrophage polarization induced by GC cell-derived exosomal circGLIS3 could enhance the invasion and metastasis of GC cells.

### The expression of circGLIS3 in GC is regulated by EIF4A3

**Fig. 8:**
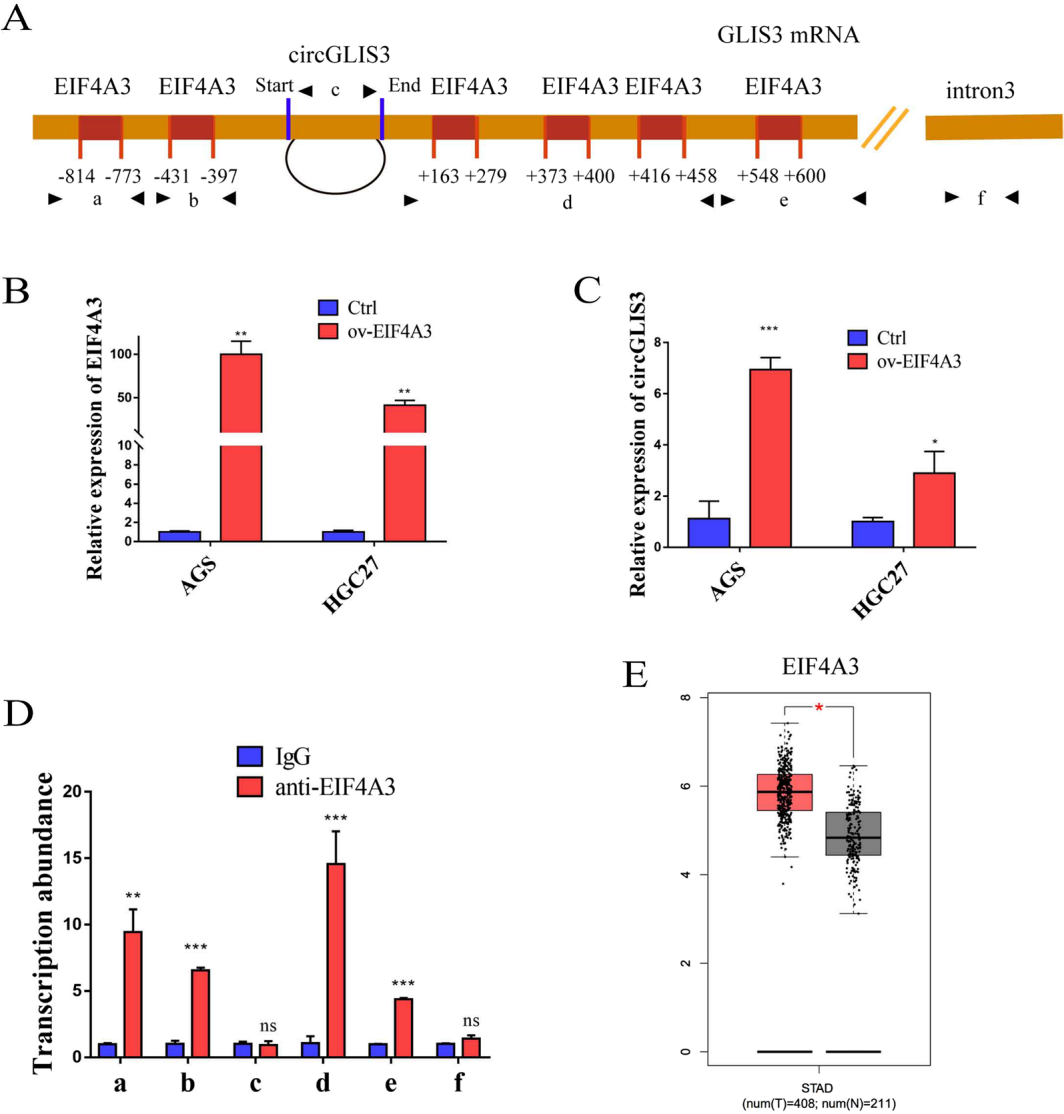
EIF4A3 regulates the generation of circGLIS3. **A**. Schematic diagram of upstream and downstream binding sites of the CircInteractome database EIF4A3 and circGLIS3. **B**. Overexpression of EIF4A3 efficiency. **C**. Changes in circGLIS3 after overexpression of EIF4A3. **D**. RIP experiment verified that EIF4A3 was combined with the upstream and downstream sequences of circGLIS3. **E**. Higher expression of EIF4A3 in gastric cancer tissue compared with normal tissue according to the TCGA database. * P < 0.05, **P < 0.01, ***P < 0.001

CircRNA formation has been reported to be regulated by RNA helicases [28, 29]. According to the CircInteractome database, six EIF4A3 (DDX48) binding sites were identified in the upstream and downstream sequences of the circGLIS3 mRNA transcript (Fig. 8A), suggesting that the formation of circGLIS3 is controlled by EIF4A3. When we overexpressed EIF4A3 in gastric cancer cells, the expression of circGLIS3 increased (Fig. 8B, C), which proved that EIF4A3 could indeed promote the generation of circGLIS3. By designing primers for EIF4A3 binding to different segments of circGLIS3 upstream and downstream sequences, we proved that EIF4A3 can bind with GLIS3 mRNA through the six putative binding sites, which we named a, b, d and e, but not circGLIS3 and intron 3 (we named c and f) using RIP experiments (Fig. 8D). EIF4A3 is significantly upregulated in gastric cancer (Fig. 8E) and has been reported to play a tumor-promoting role in a variety of tumors. The mechanism by which circGLIS3 is regulated by EIF4A3 provides a comprehensive understanding of the pathogenesis of gastric cancer. In summary, the upregulation of circGLIS3 was attributed at least in part to the promotion of EIF4A3 in GC tissues.

## Discussion

New evidence shows that circRNAs are closely related to various diseases, especially cancer [30–32]. The stable structure and biological characteristics of circRNAs have made them a new hotspot in biomedical research in recent years. In this study, we identified circGLIS3 by circRNA sequencing and confirmed that circGLIS3 was significantly upregulated in gastric cancer tissues and cell lines. In terms of function, circGLIS3 plays a role in promoting GC progression by sponging the tumor suppressor miR-1343-3p and regulating the phosphorylation of VIMENTIN Ser83. Therefore, our findings indicate that circGLIS3 can be used as a potential clinical diagnostic and therapeutic target for GC.

Increasing evidence has shown that circRNAs play a variety of biological functions by acting as miRNA sponges. As competitive endogenous RNAs (ceRNAs), circRNAs regulate miRNA function by inhibiting the binding of miRNAs to their target 3’UTRs. In our study, we explored the regulatory role of circGLIS3 in GC progression. First, we combined pull-down sequencing followed by miRNA-seq with bioinformatics prediction to verify whether miR-1343-3p is the binding miRNA of circGLIS3. Subsequently, we utilized a dual-luciferase assay to verify their complementary binding. Many researchers have reported that miR-1343-3p has antitumor effects in a variety of tumors, as well as the regulatory effect of miR-1343-3p on PGK1. In addition, the activation of PGK1 on the PI3K/AKT signaling pathway has been widely reported [23, 33]. In the next phase, the regulatory effect of circGLIS3 and miR-1343-3p on GC biological behavior through regulating PGK1 expression and activating the PI3K/AKT signaling pathway was proven by rescue experiments and a series of functional experiments. Thus, the mechanism of the circGLIS3/miR-1343-3p/PGK1 axis in gastric cancer progression was determined.

CircRNAs can also regulate tumorigenesis and development by interacting with proteins. The EMT process has been recognized as a biological cell reprogramming process characterized by loss of cell adhesion [34]. Among them, phosphorylation and structural rearrangement of different VIMENTIN sites play an important role in the EMT process, and enhanced phosphorylation of the VIMENTINser83 site can inhibit the invasion and migration of gastric cancer [35]. Therefore, exploring the specific mechanism of different phosphorylation sites of VIMENTIN can improve the theoretical basis of gastric cancer metastasis. According to our experiments and analysis, circGLIS3 overexpression can reduce the phosphorylation of VIMENTIN at Ser83, revealing the specific mechanism by which circGLIS3 reduces the phosphorylation level of VIMENTIN to promote the invasion and metastasis of gastric cancer.

In the process of tumor metastasis, tumor cells reshape the premetastatic microenvironment by regulating different phenotypes of macrophages and fibroblasts via exosome communication [36–38]. Effective intervention in this process can provide new strategies for tumor metastases. Tumor-derived exosomal circRNAs have been verified to induce tumor-associated macrophage TAM polarization to the M2 type in a variety of tumors, thereby mediating the process of tumor invasion and metastasis [39, 40]. In our study, we first proved that circGLIS3 could be transmitted by exosomal communication between GC cells and that exosomal circGLIS3 could further promote tumor metastasis in vivo. In addition, exosomal circGLIS3 promotes the polarization of macrophages to the M2 type, and exosomal circGLIS3-polarized M2-type macrophages promote gastric cancer metastasis. We revealed that exosomal circGLIS3 can not only mediate communication between gastric cancer cells but also between gastric cancer cells and macrophages, thus promoting the progression of gastric cancer.

The RNA helicase EIF4A3 can regulate exon connection and is the core component of the junction complex, which plays an important role in pre-mRNA splicing [41]. Studies have reported that EIF4A3 is elevated in many tumors, such as glioblastoma, hepatocellular carcinoma, pancreatic cancer and ovarian cancer [42]. Our research showed that EIF4A3 can regulate the generation of circGLIS3 by binding with the upstream and downstream sequences of circGLIS3, which is consistent with previous reports. Therefore, EIF4A3 plays an important role in the process of circGLIS3 splicing, and revealing the generation mechanism of circGLIS3 will help us better understand the deep mechanism of abnormal circRNA expression in gastric cancer.

**Fig. 9:**
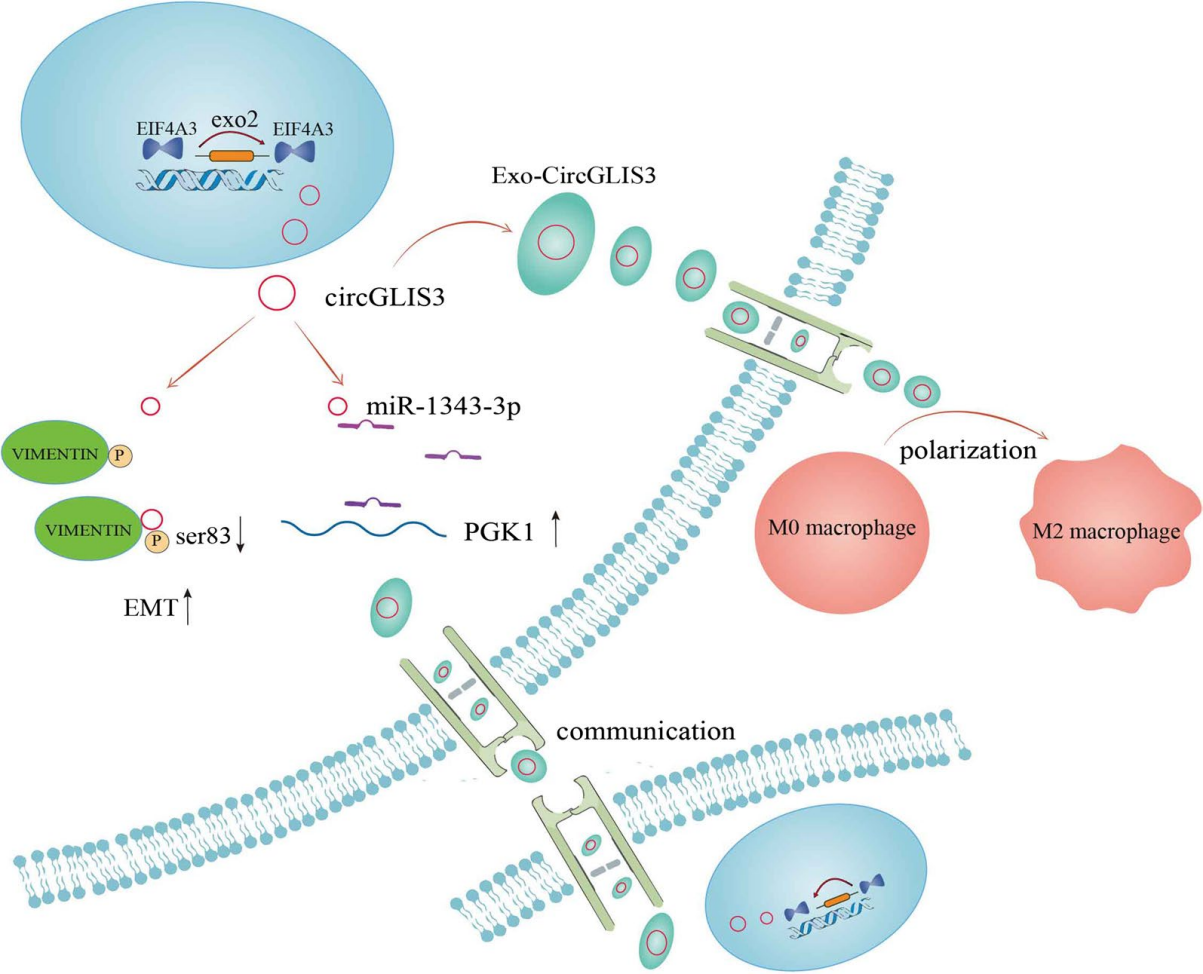
Schematic map of circGLIS3 in gastric cancer. The entire process of circGLIS3 as a tumor promoter from its generation, intracellular regulation mechanism, and cell–cell regulation in the form of exosomes. Mechanistically, circGLIS3 sponges miR-1343-3p to affect the expression level of PGK1 and reduces vimentin phosphorylation at Ser83. CircGLIS3 is packaged into exosomes and delivered to neighboring gastric cancer cells and macrophages, enabling them to acquire invasive ability and M2 macrophage properties respectively, and in turn promoting gastric cancer progression

In conclusion, we identified circGLIS3, which could be regulated by EIF4A3, to be upregulated in gastric cancer tissues and to correlate positively with gastric cancer progression. Functionally, we confirmed that circGLIS3 promotes proliferation, invasion and metastasis progression of gastric cancer in vitro and in vivo. Mechanistically, circGLIS3 sponges miR-1343-3p to affect the expression level of PGK1 and reduces vimentin phosphorylation at Ser83. Moreover, circGLIS3 is packaged into exosomes and transmitted to neighboring gastric cancer cells and macrophages, enabling them to acquire invasive capacity and M2 macrophage properties respectively, thereby promoting the progression of gastric cancer (Fig. 9). Therefore, this study clarified the expression, function and regulatory mechanism of circGLIS3 in gastric cancer and proved that circGLIS3 can be a promising target for gastric cancer treatment.

### Supplementary Information

**Additional file 1: Figure S1.** Cellular localization, overexpression and knockdown efficiency of CircGLIS3. A, B. GO and KEGG analysis of differentially expressed circRNA-derived genes. C. Expression of circGLIS3 in GES-1 and gastric cancer cell lines. D. E. The knockdown efficiency of circGLIS3. F. G. Overexpression efficiency of circGLIS3. H. I. Verification of the stable efficiency of circGLIS3 overexpression/knockdown in BGC823 cells. * P<0.05, **P<0.01, ***P<0.001. **Figure S2.** The role of circGLIS3 in promoting the growth, proliferation, invasion and metastasis of gastric cancer in the AGS cell line. A, B. CircGLIS3 overexpression significantly promoted the cell proliferation rate, as indicated by the EdU assay and the CCK8 assay. C, D. Overexpression of circGLIS3 successfully promoted the migration and invasion ability of GC cells, as determined by the Transwell experiment and wound healing assay. E.F.G.H. Nude female mice were injected with 2 × 106 stably overexpressed circGLIS3 and the corresponding control GC cells through the tail vein, and lung metastasis in vivo was evaluated using live imaging combined with HE staining. I.J.K. Nude female mice were subcutaneously injected with 5 × 106 stably overexpressed circGLIS3 and the corresponding control GC cells, and the tumors were extracted after 21 days. *P<0.05, **P<0.01, ***P<0.001, ****P<0.0001. **Figure S3.** MiR-1343-3p and PGK1 as downstream targets of circGLIS3. A. Cotransfection of circGLIS3-MS2GFP and MS2-CP-FlagmCherry plasmids to induce the expression of MS2 RNA hairpins with overexpressed circGLIS3 and a fusion protein MS2-CP-Flag, which could recognize MS2 RNA hairpins (Scale bar = 200 μm). Green fluorescence-labeled circGLIS3 (middle) and red fluorescence-labeled MS2-CP-Flag (right). B, C. ceRNA network construction and KEGG analysis of miRNAs pulled down by circGLIS3. D. The expression of hsa-miR-1343-3p was measured by qRT‒PCR after transfection with the circGLIS3 overexpression vector. E. KEGG analysis of the miRNAs pulled down by circGLIS3. F. The binding site of hsa-miR-1343-3p and PGK1. G. Changes in PGK1 protein after knockdown of circGLIS3. MS2-CP: MS2 bacteriophage coat protein, MS: mass spectrometric, GFP: green fluorescent protein, *P＜0.05, **P＜0.01, ***P＜0.001. **Figure S4.** Verification of the circGLIS3/miR-1343-3p/PGK1 axis in the AGS cell line by rescue experiments. A, B. The expression levels of PGK1 mRNA and protein and the AKT signaling pathway after overexpression of circGLIS3 and miR-1343-3p were measured by qRT‒PCR and WB in AGS cell lines. C. Cotransfection of miR-1343-3p mimics and circGLIS3 overexpression plasmid to investigate malignant transformation of cells with Transwell (C), wound healing (D), and EdU assays (E) in the AGS cell line* P<0.05, **P<0.01, ***P<0.001, ****P<0.0001. **Figure S5.** Exosomal circGLIS3 promotes the progression of gastric cancer cells. A. qRT‒PCR was used to verify the expression of circGLIS3 in the exosomes of circGLIS3-overexpressing GC cells and NC cells. B. We used a transmission electron microscope (TEM) to determine the existence and morphology of exosomes purified from GC cell medium (exosome-free FBS). C. Red exosome signals were found in the cytoplasm of GC cells when purified exosomes were added to phalloidin-labeled GC cells for 72 h. D. We detected higher circGLIS3 expression in GC cells by coculturing them with exosomes from circGLIS3-overexpressing GC cells compared with NC cells for 72 h via qRT‒PCR. E. WB was used to verify the changes in PGK1, p-AKT, p-PI3K, and vimentin Ser83 in GC cells after coculturing them with exosomes purified from circGLIS3-overexpressing GC cells relative to those from NC cells. F. G. We found that GC cells treated with circGLIS3-overexpressing exosomes showed higher metastatic and proliferative potential. M.N. Nude female mice were injected with exosomes purified from overexpressed circGLIS3 and the corresponding control GC cells through the tail vein, and lung metastasis in vivo was evaluated using live imaging combined with HE staining. * P<0.05, **P<0.01, ***P<0.001, ****P<0.0001.

**Additional file 2. **RNA-seq analysis from 3 gastric cancer tissues and their paired adjacent normal tissues.

**Additional file 3. **MiRNA sequencing pulled down by circGLIS3.

**Additional file 4. **MiRNAs with binding ability to circGLIS3 predicted by circBank database.

**Additional file 5. **Potential target gene of miR-1343-3p using multiple databases.

**Additional file 6. **KEGG analysis of the predicted downstream genes of miR-1343-3p.

**Additional file 7. **PGK1 was identified as the downstream target of miR-1343-3p by ENCORI database.

**Additional file 8. **Peptides of proteins bound to circGLIS3 detected by mass spectrometry.

## Data Availability

The datasets presented in this study can be found in online repositories. The names of the repository/repositories and accession number(s) can be found below: Gene Expression Omnibus, GSE193453.

## References

[1] Siegel RL, Miller KD, Jemal A (2019). Cancer statistics, 2019. CA Cancer J Clin.

[2] Luo Z, Rong Z, Huang C (2019). Surgery strategies for gastric cancer with liver metastasis. Front Oncol.

[3] Cancer Genome Atlas Research N (2014). Comprehensive molecular characterization of gastric adenocarcinoma. Nature.

[4] Vo JN, Cieslik M, Zhang Y, Shukla S, Xiao L, Zhang Y (2019). The landscape of circular RNA in cancer. Cell.

[5] Guarnerio J, Bezzi M, Jeong JC, Paffenholz SV, Berry K, Naldini MM (2016). Oncogenic role of fusion-circRNAs derived from cancer-associated chromosomal translocations. Cell.

[6] Qu S, Yang X, Li X, Wang J, Gao Y, Shang R (2015). Circular RNA: a new star of noncoding RNAs. Cancer Lett.

[7] Zhang M, Zhao K, Xu X, Yang Y, Yan S, Wei P (2018). A peptide encoded by circular form of LINC-PINT suppresses oncogenic transcriptional elongation in glioblastoma. Nat Commun.

[8] Yu J, Xu QG, Wang ZG, Yang Y, Zhang L, Ma JZ (2018). Circular RNA cSMARCA5 inhibits growth and metastasis in hepatocellular carcinoma. J Hepatol.

[9] Ding L, Zhao Y, Dang S, Wang Y, Li X, Yu X (2019). Circular RNA circ-DONSON facilitates gastric cancer growth and invasion via NURF complex dependent activation of transcription factor SOX4. Mol Cancer.

[10] Hsiao KY, Lin YC, Gupta SK, Chang N, Yen L, Sun HS (2017). Noncoding effects of circular RNA CCDC66 promote colon cancer growth and metastasis. Cancer Res.

[11] Ren GL, Zhu J, Li J, Meng XM (2019). Noncoding RNAs in acute kidney injury. J Cell Physiol.

[12] Wang Y, Liu J, Ma J, Sun T, Zhou Q, Wang W (2019). Exosomal circRNAs: biogenesis, effect and application in human diseases. Mol Cancer.

[13] Wang Y, Gao R, Li J, Tang S, Li S, Tong Q (2021). Downregulation of hsa_circ_0074854 suppresses the migration and invasion in hepatocellular carcinoma via interacting with HuR and via suppressing exosomes-mediated macrophage M2 polarization. Int J Nanomedicine.

[14] Aucher A, Rudnicka D, Davis DM (2013). MicroRNAs transfer from human macrophages to hepato-carcinoma cells and inhibit proliferation. J Immunol.

[15] Szebeni GJ, Vizler C, Kitajka K, Puskas LG (2017). Inflammation and cancer: extra- and intracellular determinants of tumor-associated macrophages as tumor promoters. Mediators Inflamm.

[16] Kim J, Bae JS (2016). Tumor-associated macrophages and neutrophils in tumor microenvironment. Mediators Inflamm.

[17] Qian BZ, Pollard JW (2010). Macrophage diversity enhances tumor progression and metastasis. Cell.

[18] Noy R, Pollard JW (2014). Tumor-associated macrophages: from mechanisms to therapy. Immunity.

[19] Lin B, Chen X, Lu C, Xu J, Qiu Y, Liu X (2021). Loss of exosomal LncRNA HCG15 prevents acute myocardial ischemic injury through the NF-kappaB/p65 and p38 pathways. Cell Death Dis.

[20] Han K, Wang FW, Cao CH, Ling H, Chen JW, Chen RX (2020). CircLONP2 enhances colorectal carcinoma invasion and metastasis through modulating the maturation and exosomal dissemination of microRNA-17. Mol Cancer.

[21] Gilani N, Arabi Belaghi R, Aftabi Y, Faramarzi E, Edgunlu T, Somi MH (2021). Identifying potential miRNA biomarkers for gastric cancer diagnosis using machine learning variable selection approach. Front Genet.

[22] Zhou Y, Huang T, Zhang J, Wong CC, Zhang B, Dong Y (2017). TEAD1/4 exerts oncogenic role and is negatively regulated by miR-4269 in gastric tumorigenesis. Oncogene.

[23] Wang L, Bo X, Yi X, Xiao X, Zheng Q, Ma L (2020). Exosome-transferred LINC01559 promotes the progression of gastric cancer via PI3K/AKT signaling pathway. Cell Death Dis.

[24] Yamaguchi T, Goto H, Yokoyama T, Sillje H, Hanisch A, Uldschmid A (2005). Phosphorylation by Cdk1 induces Plk1-mediated vimentin phosphorylation during mitosis. J Cell Biol.

[25] Xie M, Yu T, Jing X, Ma L, Fan Y, Yang F (2020). Exosomal circSHKBP1 promotes gastric cancer progression via regulating the miR-582-3p/HUR/VEGF axis and suppressing HSP90 degradation. Mol Cancer.

[26] Zhang PF, Gao C, Huang XY, Lu JC, Guo XJ, Shi GM (2020). Cancer cell-derived exosomal circUHRF1 induces natural killer cell exhaustion and may cause resistance to anti-PD1 therapy in hepatocellular carcinoma. Mol Cancer.

[27] Shang A, Gu C, Wang W, Wang X, Sun J, Zeng B (2020). Exosomal circPACRGL promotes progression of colorectal cancer via the miR-142-3p/miR-506-3p- TGF-beta1 axis. Mol Cancer.

[28] Wang R, Zhang S, Chen X, Li N, Li J, Jia R (2018). EIF4A3-induced circular RNA MMP9 (circMMP9) acts as a sponge of miR-124 and promotes glioblastoma multiforme cell tumorigenesis. Mol Cancer.

[29] Wei Y, Lu C, Zhou P, Zhao L, Lyu X, Yin J (2021). EIF4A3-induced circular RNA ASAP1 promotes tumorigenesis and temozolomide resistance of glioblastoma via NRAS/MEK1/ERK1-2 signaling. Neuro Oncol.

[30] Chen J, Li Y, Zheng Q, Bao C, He J, Chen B (2017). Circular RNA profile identifies circPVT1 as a proliferative factor and prognostic marker in gastric cancer. Cancer Lett.

[31] Dang Y, Ouyang X, Zhang F, Wang K, Lin Y, Sun B (2017). Circular RNAs expression profiles in human gastric cancer. Sci Rep.

[32] Rong D, Lu C, Zhang B, Fu K, Zhao S, Tang W (2019). CircPSMC3 suppresses the proliferation and metastasis of gastric cancer by acting as a competitive endogenous RNA through sponging miR-296-5p. Mol Cancer.

[33] Zieker D, Konigsrainer I, Tritschler I, Loffler M, Beckert S, Traub F (2010). Phosphoglycerate kinase 1 a promoting enzyme for peritoneal dissemination in gastric cancer. Int J Cancer.

[34] Paolillo M, Schinelli S (2019). Extracellular matrix alterations in metastatic processes. Int J Mol Sci.

[35] Zeng S, Xie X, Xiao YF, Tang B, Hu CJ, Wang SM (2018). Long noncoding RNA LINC00675 enhances phosphorylation of vimentin on Ser83 to suppress gastric cancer progression. Cancer Lett.

[36] Li W, Ng JM, Wong CC, Ng EKW, Yu J (2018). Molecular alterations of cancer cell and tumour microenvironment in metastatic gastric cancer. Oncogene.

[37] Li Z, Yanfang W, Li J, Jiang P, Peng T, Chen K (2018). Tumor-released exosomal circular RNA PDE8A promotes invasive growth via the miR-338/MACC1/MET pathway in pancreatic cancer. Cancer Lett.

[38] Li J, Li Z, Jiang P, Peng M, Zhang X, Chen K (2018). Circular RNA IARS (circ-IARS) secreted by pancreatic cancer cells and located within exosomes regulates endothelial monolayer permeability to promote tumor metastasis. J Exp Clin Cancer Res.

[39] Wu K, Lin K, Li X, Yuan X, Xu P, Ni P (2020). Redefining tumor-associated macrophage subpopulations and functions in the tumor microenvironment. Front Immunol.

[40] Mousavi S, Moallem R, Hassanian SM, Sadeghzade M, Mardani R, Ferns GA (2019). Tumor-derived exosomes: potential biomarkers and therapeutic target in the treatment of colorectal cancer. J Cell Physiol.

[41] Zheng X, Huang M, Xing L, Yang R, Wang X, Jiang R (2020). The circRNA circSEPT9 mediated by E2F1 and EIF4A3 facilitates the carcinogenesis and development of triple-negative breast cancer. Mol Cancer.

[42] Ye J, She X, Liu Z, He Z, Gao X, Lu L (2021). Eukaryotic initiation factor 4A–3: a review of its physiological role and involvement in oncogenesis. Front Oncol.

