# Supplementary material for: CircGLIS3 promotes gastric cancer progression by regulating the miR-1343-3p/PGK1 pathway and inhibiting vimentin phosphorylation
Source: J Transl Med. 2024 Mar 8;22:251. doi: 10.1186/s12967-023-04625-2 (PMC10921581; doi:10.1186/s12967-023-04625-2)
Supplement: Supplementary file 1 — Additional file 1: Figure S1. Cellular localization, overexpression and knockdown efficiency of CircGLIS3. A, B. GO and KEGG analysis of differentially expressed circRNA-derived genes. C. Expression of circGLIS3 in GES-1 and gastric cancer cell lines. D. E. The knockdown efficiency of circGLIS3. F. G. Overexpression efficiency of circGLIS3. H. I. Verification of the stable efficiency of circGLIS3 overexpression/knockdown in BGC823 cells. * P<0.05, **P<0.01, ***P<0.001. Figure S2. The role of circGLIS3 in promoting the growth, proliferation, invasion and metastasis of gastric cancer in the AGS cell line. A, B. CircGLIS3 overexpression significantly promoted the cell proliferation rate, as indicated by the EdU assay and the CCK8 assay. C, D. Overexpression of circGLIS3 successfully promoted the migration and invasion ability of GC cells, as determined by the Transwell experiment and wound healing assay. E.F.G.H. Nude female mice were injected with 2 × 10^6^ stably overexpressed circGLIS3 and the corresponding control GC cells through the tail vein, and lung metastasis in vivo was evaluated using live imaging combined with HE staining. I.J.K. Nude female mice were subcutaneously injected with 5 × 10^6^ stably overexpressed circGLIS3 and the corresponding control GC cells, and the tumors were extracted after 21 days. *P<0.05, **P<0.01, ***P<0.001, ****P<0.0001. Figure S3. MiR-1343-3p and PGK1 as downstream targets of circGLIS3. A. Cotransfection of circGLIS3-MS2GFP and MS2-CP-FlagmCherry plasmids to induce the expression of MS2 RNA hairpins with overexpressed circGLIS3 and a fusion protein MS2-CP-Flag, which could recognize MS2 RNA hairpins (Scale bar = 200 μm). Green fluorescence-labeled circGLIS3 (middle) and red fluorescence-labeled MS2-CP-Flag (right). B, C. ceRNA network construction and KEGG analysis of miRNAs pulled down by circGLIS3. D. The expression of hsa-miR-1343-3p was measured by qRT‒PCR after transfection with the circGLIS3 overexpression vector. E. KEGG anal [file 12967_2023_4625_MOESM1_ESM.docx]

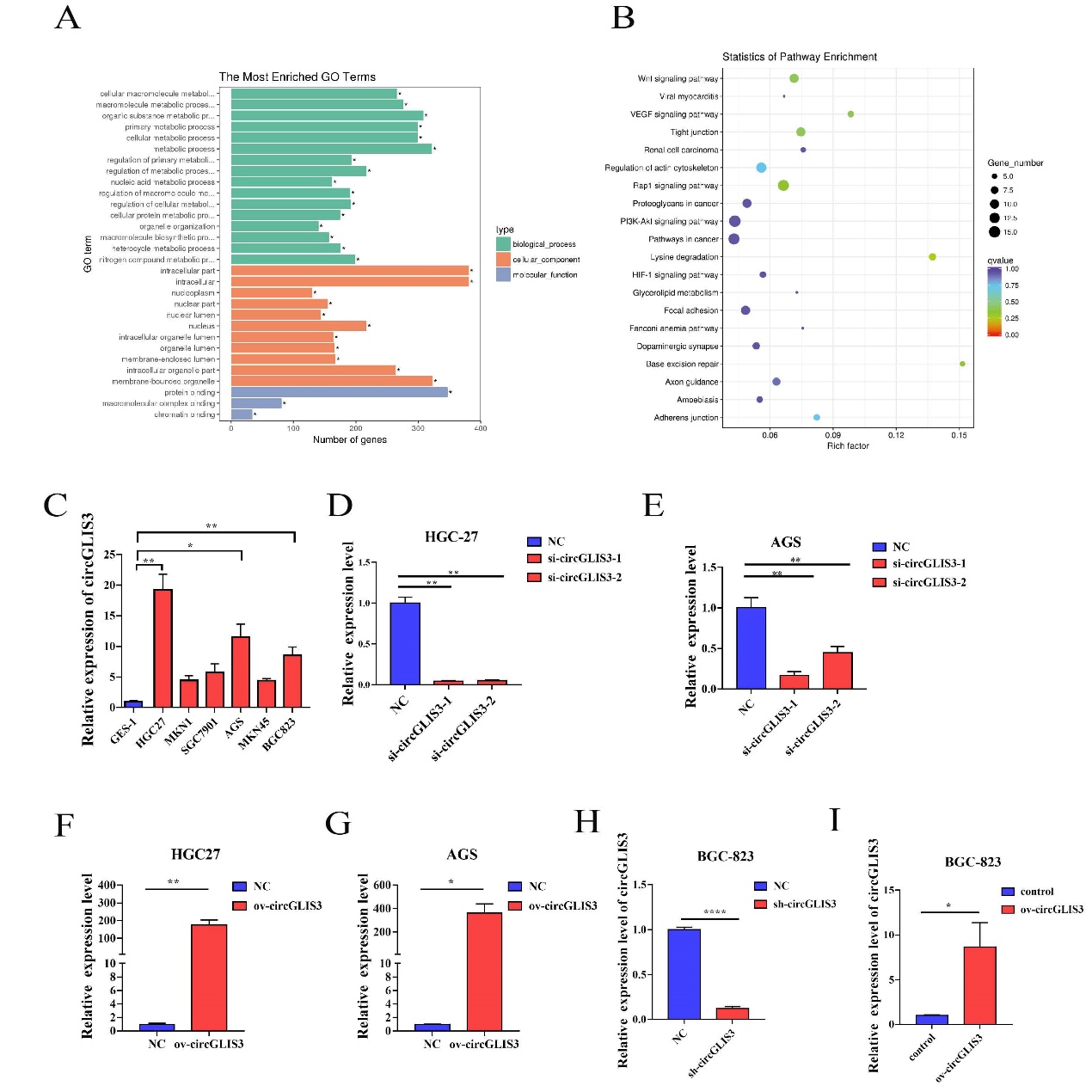
**Figure S1 Cellular localization, overexpression and knockdown efficiency of CircGLIS3**

A, B. GO and KEGG analysis of differentially expressed circRNA-derived genes. C. Expression of circGLIS3 in GES-1 and gastric cancer cell lines. D. E. The knockdown efficiency of circGLIS3. F. G. Overexpression efficiency of circGLIS3. H. I. Verification of the stable efficiency of circGLIS3 overexpression/knockdown in BGC823 cells. * P<0.05, **P<0.01, ***P<0.001.


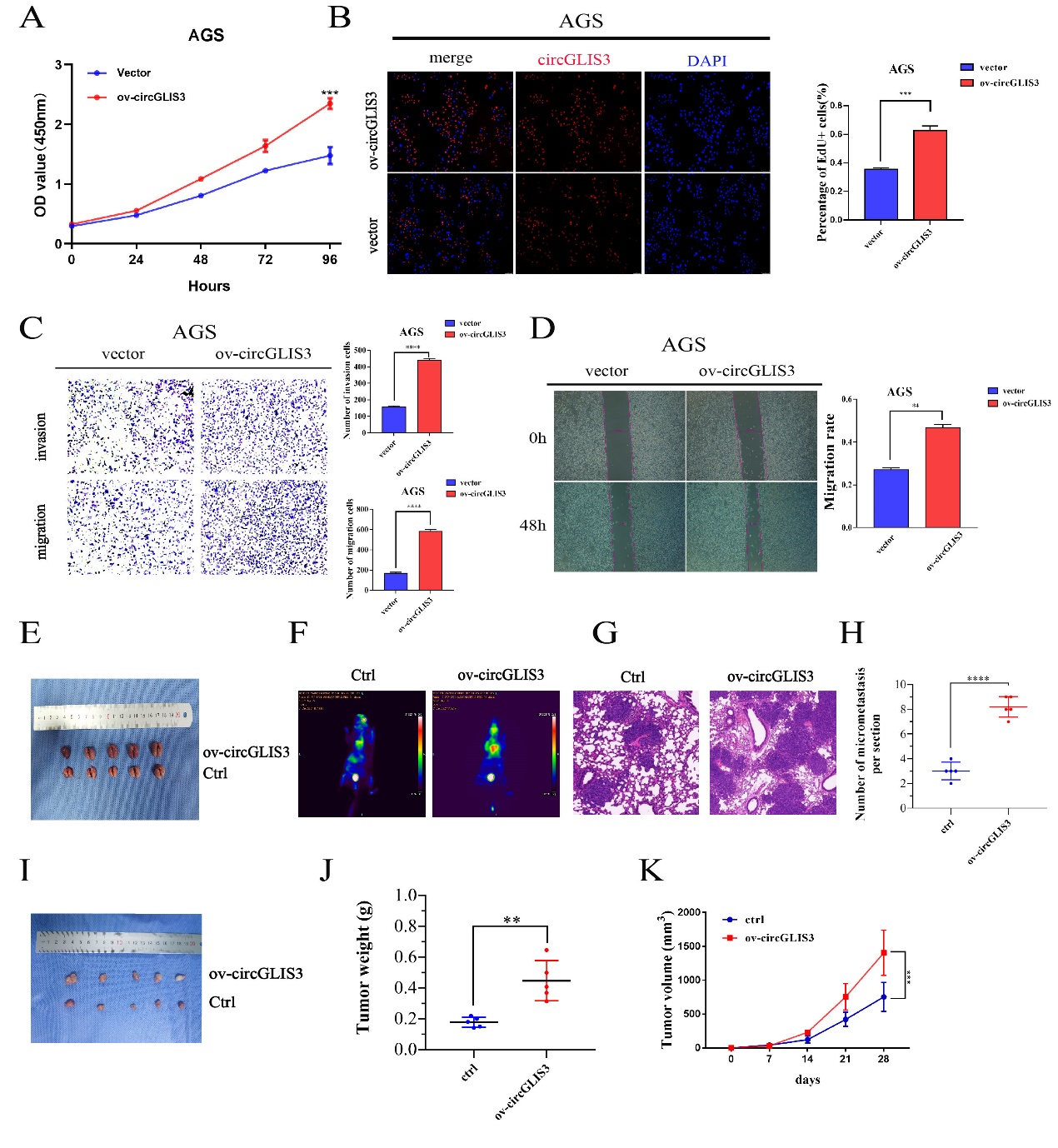


**Figure S2 The role of circGLIS3 in promoting the growth, proliferation, invasion and metastasis of gastric cancer in the AGS cell line.**

A, B. CircGLIS3 overexpression significantly promoted the cell proliferation rate, as indicated by the EdU assay and the CCK8 assay. C, D. Overexpression of circGLIS3 successfully promoted the migration and invasion ability of GC cells, as determined by the Transwell experiment and wound healing assay. E.F.G.H. Nude female mice were injected with 2 × 10^6^ stably overexpressed circGLIS3 and the corresponding control GC cells through the tail vein, and lung metastasis in vivo was evaluated using live imaging combined with HE staining. I.J.K. Nude female mice were subcutaneously injected with 5 × 10^6^ stably overexpressed circGLIS3 and the corresponding control GC cells, and the tumors were extracted after 21 days. *P<0.05, **P<0.01, ***P<0.001, ****P<0.0001.


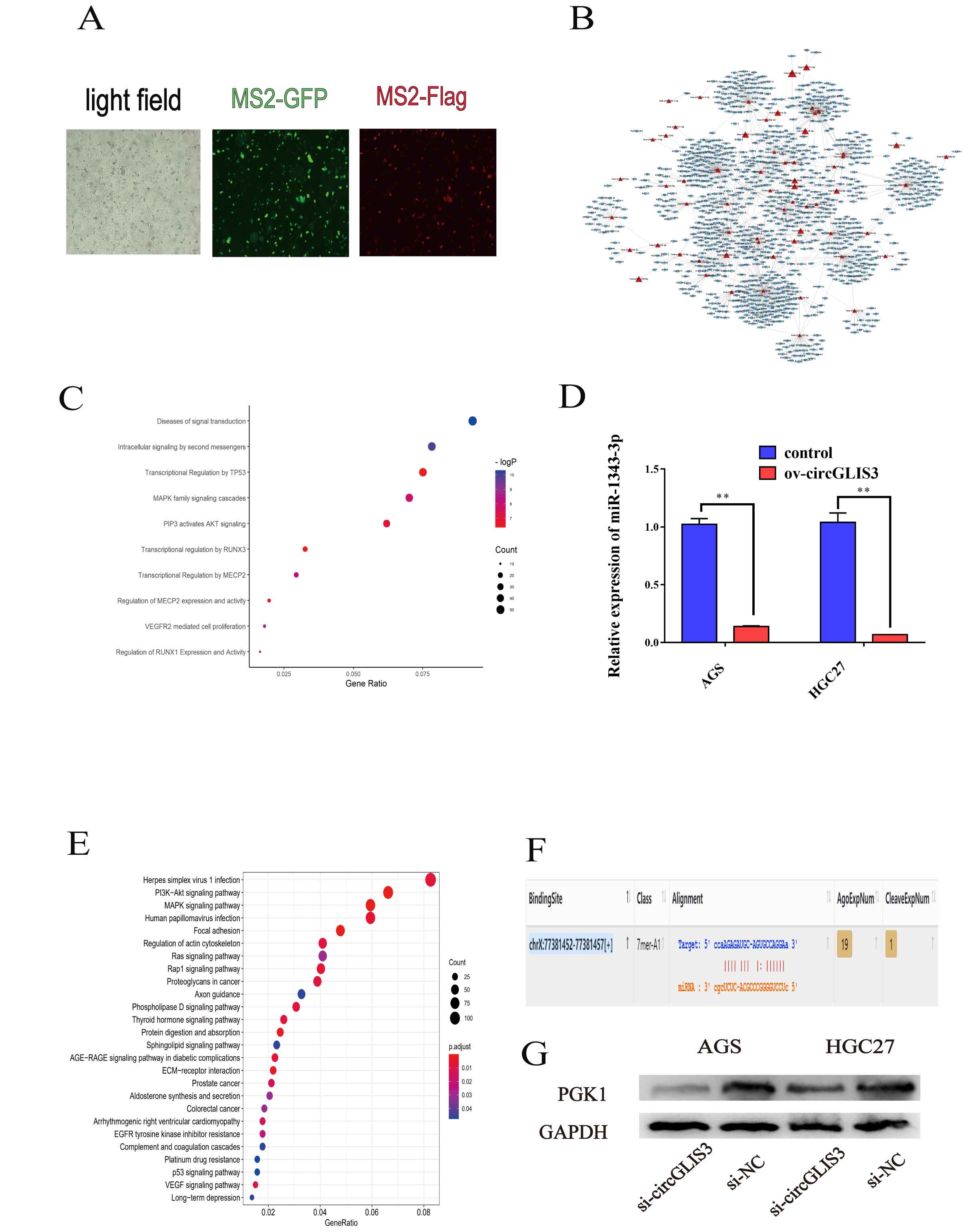


**Figure S3 MiR-1343-3p and PGK1 as downstream targets of circGLIS3**

A. Cotransfection of circGLIS3-MS2GFP and MS2-CP-FlagmCherry plasmids to induce the expression of MS2 RNA hairpins with overexpressed circGLIS3 and a fusion protein MS2-CP-Flag, which could recognize MS2 RNA hairpins (Scale bar = 200 μm). Green fluorescence-labeled circGLIS3 (middle) and red fluorescence-labeled MS2-CP-Flag (right). B, C. ceRNA network construction and KEGG analysis of miRNAs pulled down by circGLIS3. D. The expression of hsa-miR-1343-3p was measured by qRT‒PCR after transfection with the circGLIS3 overexpression vector. E. KEGG analysis of the miRNAs pulled down by circGLIS3. F. The binding site of hsa-miR-1343-3p and PGK1. G. Changes in PGK1 protein after knockdown of circGLIS3. MS2-CP: MS2 bacteriophage coat protein, MS: mass spectrometric, GFP: green fluorescent protein, *P＜0.05, **P＜0.01, ***P＜0.001.


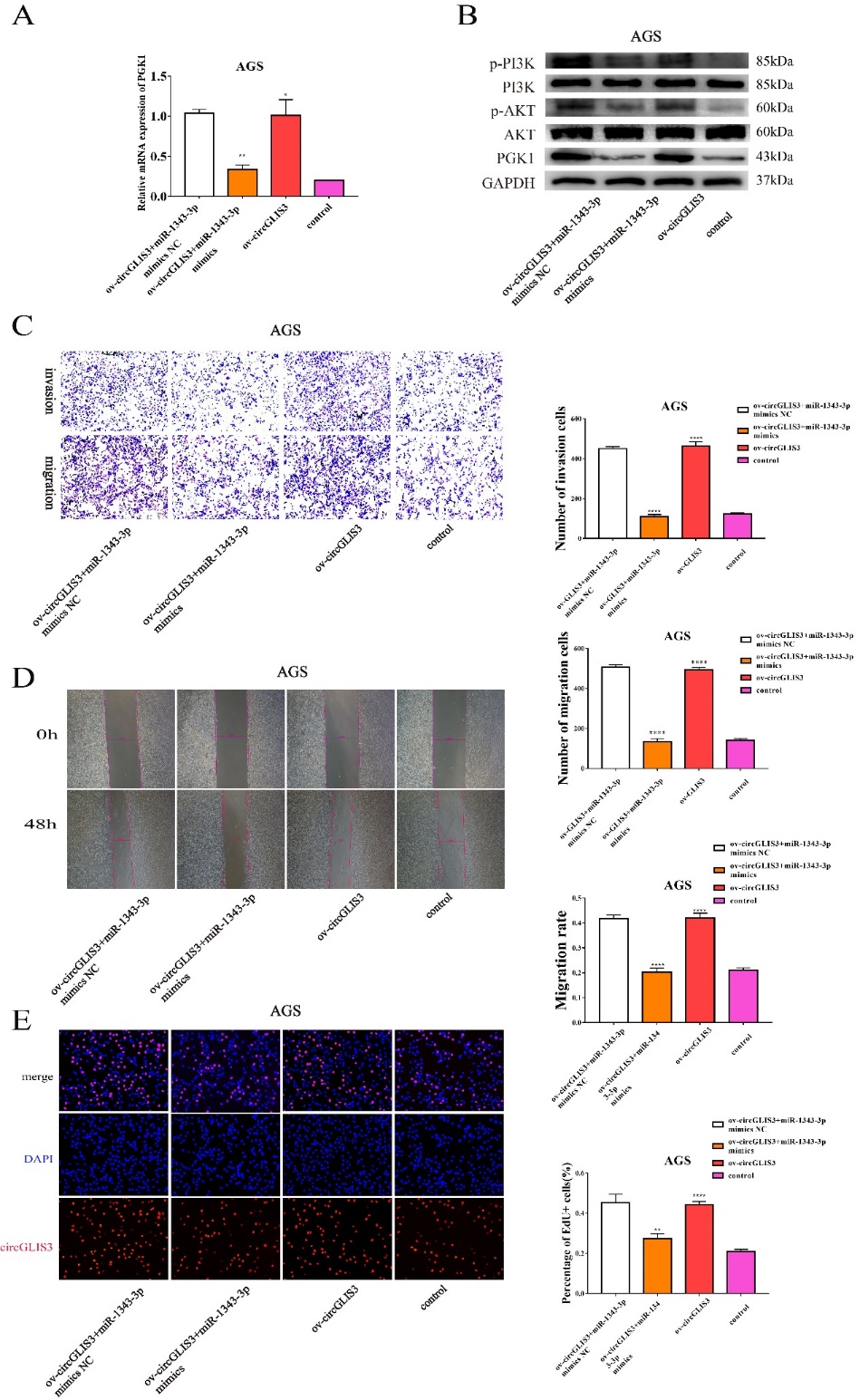


**Figure S4 Verification of the circGLIS3/miR-1343-3p/PGK1 axis in the AGS cell line by rescue experiments**

A, B. The expression levels of PGK1 mRNA and protein and the AKT signaling pathway after overexpression of circGLIS3 and miR-1343-3p were measured by qRT‒PCR and WB in AGS cell lines. C. Cotransfection of miR-1343-3p mimics and circGLIS3 overexpression plasmid to investigate malignant transformation of cells with Transwell (C), wound healing (D), and EdU assays (E) in the AGS cell line* P<0.05, **P<0.01, ***P<0.001, ****P<0.0001.


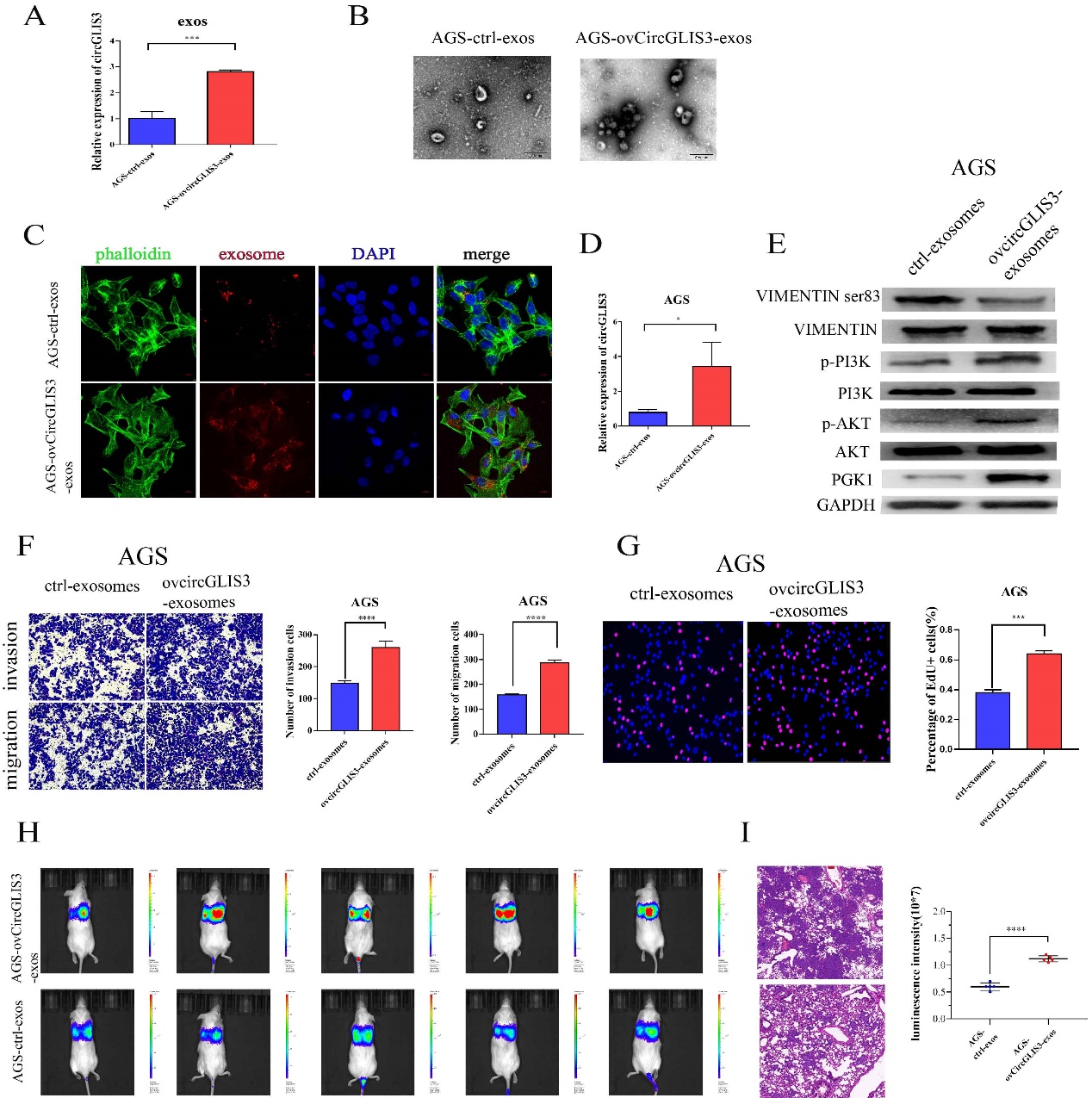


**Figure S5 Exosomal circGLIS3 promotes the progression of gastric cancer cells**

A. qRT‒PCR was used to verify the expression of circGLIS3 in the exosomes of circGLIS3-overexpressing GC cells and NC cells. B. We used a transmission electron microscope (TEM) to determine the existence and morphology of exosomes purified from GC cell medium (exosome-free FBS). C. Red exosome signals were found in the cytoplasm of GC cells when purified exosomes were added to phalloidin-labeled GC cells for 72 h. D. We detected higher circGLIS3 expression in GC cells by coculturing them with exosomes from circGLIS3-overexpressing GC cells compared with NC cells for 72 h via qRT‒PCR. E. WB was used to verify the changes in PGK1, p-AKT, p-PI3K, and vimentin Ser83 in GC cells after coculturing them with exosomes purified from circGLIS3-overexpressing GC cells relative to those from NC cells. F. G. We found that GC cells treated with circGLIS3-overexpressing exosomes showed higher metastatic and proliferative potential. M.N. Nude female mice were injected with exosomes purified from overexpressed circGLIS3 and the corresponding control GC cells through the tail vein, and lung metastasis in vivo was evaluated using live imaging combined with HE staining. * P<0.05, **P<0.01, ***P<0.001, ****P<0.0001.
